# Blocking of counter-partisan accounts drives political assortment on Twitter

**DOI:** 10.1093/pnasnexus/pgae161

**Published:** 2024-04-15

**Authors:** Cameron Martel, Mohsen Mosleh, Qi Yang, Tauhid Zaman, David G Rand

**Affiliations:** Sloan School of Management, Massachusetts Institute of Technology, Cambridge, MA 02142, USA; Sloan School of Management, Massachusetts Institute of Technology, Cambridge, MA 02142, USA; Management Department, University of Exeter Business School, Exeter EX4 4PU, UK; Institute for Data, Systems, and Society (IDSS), Massachusetts Institute of Technology, Cambridge, MA 02142, USA; Yale School of Management, Yale University, New Haven, CT 06511, USA; Sloan School of Management, Massachusetts Institute of Technology, Cambridge, MA 02142, USA; Institute for Data, Systems, and Society (IDSS), Massachusetts Institute of Technology, Cambridge, MA 02142, USA; Department of Brain and Cognitive Sciences, Massachusetts Institute of Technology, Cambridge, MA 02139, USA

**Keywords:** homophily, social ties, blocking, social media, polarization

## Abstract

There is strong political assortment of Americans on social media networks. This is typically attributed to preferential tie formation (i.e. homophily) among those with shared partisanship. Here, we demonstrate an additional factor beyond homophily driving assorted networks: preferential *prevention* of social ties. In two field experiments on Twitter, we created human-looking bot accounts that identified as Democrats or Republicans, and then randomly assigned users to be followed by one of these accounts. In addition to preferentially following-back copartisans, we found that users were 12 times more likely to block counter-partisan accounts compared to copartisan accounts in the first experiment, and 4 times more likely to block counter-partisan accounts relative to a neutral account or a copartisan account in the second experiment. We then replicated these findings in a survey experiment and found evidence of a key motivation for blocking: wanting to avoid seeing any content posted by the blocked user. Additionally, we found that Democrats preferentially blocked counter-partisans more than Republicans, and that this asymmetry was likely due to blocking accounts who post low-quality or politically slanted content (rather than an asymmetry in identity-based blocking). Our results demonstrate that preferential blocking of counter-partisans is an important phenomenon driving political assortment on social media.

**Significance Statement**

Americans are more likely to be socially connected with copartisans. How and why does this come to be? Prior research has largely focused on preferential tie formation, documenting how people are more likely to reciprocate ties with copartisans. Here, we demonstrate another important force behind political segmentation—preferential *prevention* of social ties with counter-partisans. Across two Twitter field experiments, individuals were far more likely to block counter-partisan than copartisan or neutral accounts. Democrats were more likely to block Republicans than vice versa—and follow-up analyses suggest this may be due to asymmetries in what partisans post online. This has implications for how we think about the formation of digital social networks: politically like-minded networks form not only from who partisans choose to connect with but also from who they actively avoid.

## Introduction

Partisan identities are of central importance to many Americans (1). Accordingly, a large body of research suggests that Americans preferentially interact with supporters of their preferred party. Offline, Americans are much more likely to have face-to-face interactions with copartisans than counter-partisans (2). Americans are also largely geographically segregated by partisanship—and such political assortment is even evident within individual cities and neighborhoods (3). Political correspondence between spouses, and between parents and their children, have also increased in recent years (4).

Similar patterns of partisan assortment have been documented online. Observational data find that Americans are more likely to have connections with copartisans on social media. On Facebook, liberals are more likely to be friends with other liberals than conservatives, and vice versa at similar rates (5). Furthermore, there have been documented high levels of political assortment in social networks on Twitter among political retweet networks (6) and social assortment among both Democrats (7) and Republicans (8, 9). Such online partisan assortment is not limited to social networks—recent work has shown that there is a high degree of ideological segregation in political news exposure on Facebook, and that in particular there exists a highly segmented, homogenously conservative information subnetwork (10). Social and informational political assortment are integral features of contemporary American society.

The aforementioned research on political clustering provides thorough observational evidence of partisan assortment among Americans. However, such data do not directly speak to the *causal* mechanisms underlying these trends. What are the mechanisms by which party-based sorting occurs? Prior work on causal effects has focused on homophily—the tendency to associate and *make* connections with similar others. For example, experimental research has shown that Americans are more likely to match with dating partners with similar political characteristics (11), prefer engaging in economic interactions with copartisans (12), and even evaluate identical residential properties more favorably when informed that they are nearby more copartisans (13). In the context of social media, a recent Twitter field experiment demonstrated a causal effect of shared partisanship on the tendency to reciprocate social ties: both Democrats and Republicans were roughly 3 times more likely to reciprocally follow-back copartisan accounts compared to counter-partisan accounts (14), and this work has been replicated using different experimental designs (15) and in non-US contexts (e.g. Brazil; Ref. (16)).

These experiments all find that the tendency to form ties with politically similar others is a driving factor of political assortment. However, affiliating with shared partisans via political homophily is not the only potential route through which segregated political networks can emerge. For instance, recent work posits that in addition to homophily, social tie decisions can also be driven by political acrophily—or the tendency to associate with those with more extreme political views (17). Furthermore, some prior research has also examined how homogenous networks can also arise via social tie *breaking*. For instance, social ties between more demographically dissimilar individuals are more likely to be dissolved over time (for review, see Ref. (18)). The elimination of ties has also been examined in research on the evolution of cooperation on networks ((19–22); for review, see Ref. (23)) and in observational data on unfriending practices on social media (24, 25).

However, the making and breaking of social ties both focus exclusively on connections that have been formed or passively not established. Here, we focus on another critical form of social tie preference which has received scant attention in the context of polarization and partisan assortment on social media—the active *prevention* of social ties. Social tie prevention entails the affirmative disallowance of a social tie from being established, or from being formed in the future. Practically, social tie prevention is an important behavior to study in the context of social media because almost all major social media platforms allow for such action through “blocking.” For example, on Twitter (now called “X”), a user may block an account to prevent that account's posts from ever appearing in their timeline, prevent that account from ever contacting or following them, and prevent that account from seeing their own posts. Whereas nonfollowed or unfollowed users may still be seen in one's timeline or have the opportunity to follow or message a user, blocked accounts are prevented from such social connections. As such, blocking is an example of social tie prevention. Therefore, in addition to following, ignoring, or unfollowing accounts, blocking other individuals is an important additional social action to monitor when studying assortment (and associated “echo chambers”) online.

Theoretically, blocking may also be a particularly important action to examine in the context of political assortment. A great deal of work on affective polarization indicates that many Americans distrust and dislike counter-partisans (1, 26). Thus, partisan users may elect to block counter-partisans as a means of expressing the negative affect they feel toward those counter-partisans. Alternatively, users may expect that counter-partisans will act in a hostile way toward them, leading them to engage in blocking as a way to proactively reduce or eliminate harassment by (27) or negative engagement with counter-partisans. Informationally, partisan users may block accounts as a means of content filtering and selective exposure (28), such that accounts may be blocked so that the focal user sees less counter-partisan posts or news stories. For instance, prior qualitative work has found that more politically active individuals are more likely to purport blocking political adversaries (29). Relatedly, survey experiments find that individuals are more likely to block counter-partisans who share misinformation than copartisans who share misinformation, suggesting that both partisan identity and the type of shared content may influence decisions to prevent social ties (30). Thus, there is theoretical reason to expect preferential prevention of ties with counter-partisans, and open questions regarding what mechanism(s) may underlie such an effect.

Despite its practical and theoretical potential importance, little prior work has experimentally examined blocking as a key microfoundation of online political assortment. One published survey experiment using Americans (30) and one working paper describing a Twitter field experiment in Brazil (16) found greater propensities for partisans to block counter-partisans than copartisans. Here, we examine whether such results hold in several large-scale field and survey experiments among American Twitter users. In doing so, we compare the magnitude of the effects of shared partisanship on blocking to the effects on social tie reciprocation to provide some evidence as to the relative importance of social tie prevention behaviors compared to tie formation behaviors. Furthermore, we investigate potential partisan asymmetries in blocking. Evidence is mixed as to whether there exist asymmetries in online social political assortment. Observationally, different methodologies and time periods suggest competing perspectives as to whether Democrats or Republicans exhibit more online homophily (7–9, 31). Causal investigations of social reciprocation among Americans on Twitter haver found that both Democrats and Republicans selectively follow-back copartisans vs. counter-partisans at similar rates (14, 15). Thus, it is unclear whether preferential blocking may be symmetric or asymmetric across parties in the US context. Finally, we investigate potential psychological mechanisms underlying selective prevention of social ties with counter-partisans. Prior work is agnostic as to whether blocking reflects derogation, protection, or information avoidance. Here, we shed light on this issue directly.

Thus, the current paper aims to provide a comprehensive series of experiments investigating the causal role of social tie prevention in partisan assortment on social media. Furthermore, we seek to provide evidence as to the underlying drivers of why individuals block other users online. We ask the following research questions: (i) Do American Twitter users demonstrate preferential social tie prevention, such that they block counter-partisans more than copartisans or politically neutral accounts? (ii) Are there partisan asymmetries in blocking between Democrats and Republicans, and if so, why might such asymmetries exist? (iii) What reasons do individuals provide for blocking other users?

**Fig. 1.**
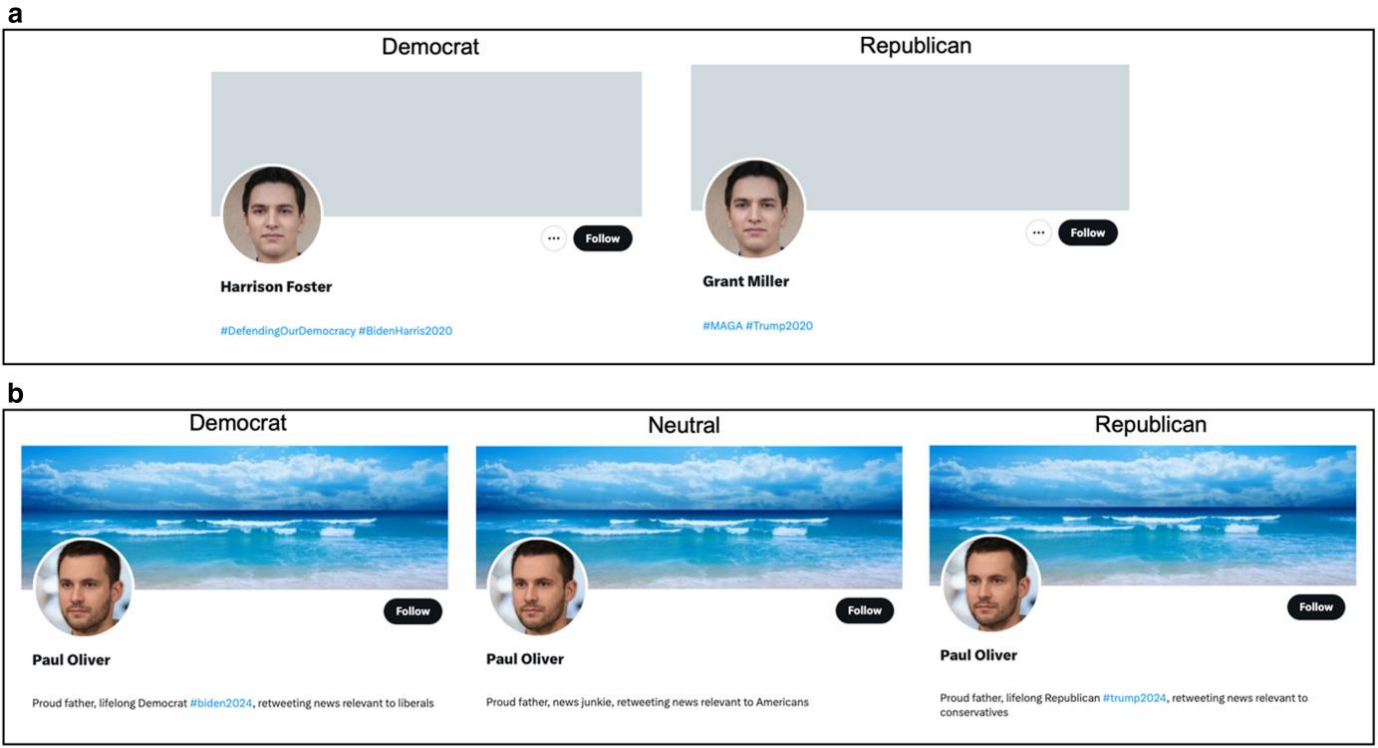
Design of field experiment bot accounts. a) Field Experiment 1 human-looking bot account examples—including Democratic and Republican profiles. Overall, we created 10 human-looking accounts (5 Republican, 5 Democrat). b) Field Experiment 2 human-looking bot account examples—including Democratic, politically neutral, and Republican bot accounts. The bot accounts in both field experiments followed a set of political elite accounts according to their political partisanship, and retweeted randomly from these accounts each day of the study. Political preferences were also indicated in profile biographies.

To examine online partisan blocking behavior, we first conducted two field experiments on Twitter (32). In Field Experiment 1, we created 10 human-looking accounts (see Fig. 1a for example accounts). Half of these accounts self-identified as favoring the Democratic Party, and half the Republican Party. To increase the credibility of our bot accounts, each account also retweeted a post from a politically aligned mainstream news outlet once every 3 days (e.g. Democratic Party accounts retweeted from “MSNBC,” “washingtonpost,” “NBCNews,” and “TheAtlantic”; Republican Party accounts retweeted from “FoxNews,” “thedispatch,” “NRO,” and “amconmag” Twitter accounts). All other account characteristics were the same between conditions. Our conditions thus jointly manipulated both the appearance and the news sharing behavior of partisan Twitter accounts. We then used these accounts to follow a politically balanced set of Twitter users and monitored whether each user blocked or followed our accounts in response. Next, we conducted Field Experiment 2, in which we deployed a similar experimental design but additionally included a politically neutral account as a control condition (see Fig. 1b for example accounts used in Field Experiment 2; neutral accounts retweeted from “Reuters,” “nprnews,” “BBCWorld,” and “AP”).

We then conducted a follow-up survey experiment to replicate our field results and shed further light on *why* users engage in blocking. In our survey experiment, we manipulated only the appearance of partisan accounts without showing news sharing behavior. We then assessed whether participants were more likely to block counter-partisan appearing profiles and what reasons they provided for blocking accounts.

## Results

### Results from Twitter field experiments

We analyze the results of Field Experiment 1 using a linear regression with a counter-partisanship dummy variable (0 = user and bot favor the same party, 1 = user and bot favor different parties), the partisanship of the user (binary measure of partisanship based on news media sharing, see Ref. (33); *z*-scored), and their interaction as predictors. We begin by replicating past findings regarding preferential tie formation (14). Consistent with past work, when predicting whether the user followed-back our bot account, we found that users were significantly less likely to follow back the counter-partisan bot compared to the copartisan bot (*b* = −0.048, SE = 0.010, *t*(2,006) = −4.905, *P* < 0.001); and that this selective follow-back behavior did not differ for Democrats and Republicans (no significant interaction between the counter-partisan dummy and the user partisanship dummy, *b* = −0.000, SE = 0.010, *t*(2,006) = −0.029, *P* = 0.977).

**Fig. 2.**
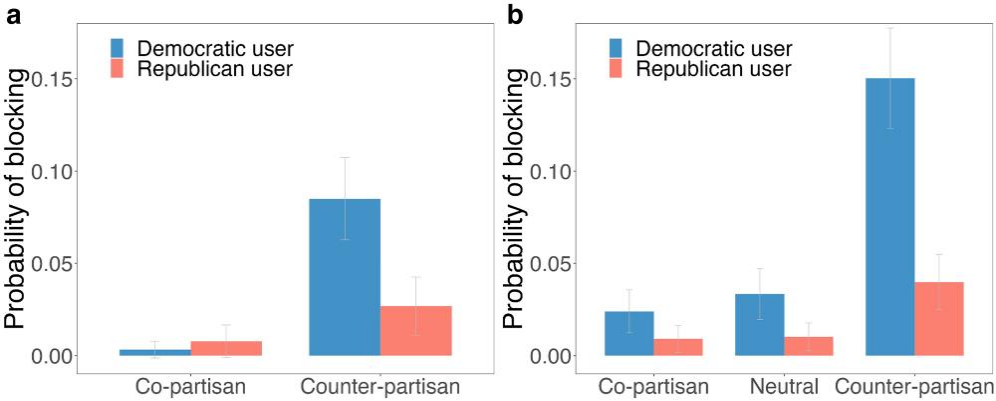
Probability of blocking by shared partisanship and user partisanship in field experiments. Error bars indicate 95% CIs. a) Results of Field Experiment 1. b) Results of Field Experiment 2.

We now turn to our key question of interest: blocking behavior. When predicting whether the user blocked our bot account, we find substantial evidence of selective blocking. Users in our sample were roughly 12 times more likely to block counter-partisan accounts compared to copartisan accounts (*b* = 0.057, SE = 0.008, *t*(2,006) = 7.247, *P* < 0.001; see Fig. 2a). Unlike for selective tie formation, we also observed a substantial partisan asymmetry in blocking rates: Democratic users were more likely to block Republican bots (26 times more likely than blocking copartisan Democrat bots) than Republican users were to block Democrat bots (about 3 times more likely than blocking copartisan Republican bots; interaction between counter-partisanship and user partisanship: *b* = −0.031, SE = 0.008, *t*(2,006) = −3.893, *P* < 0.001). Together, these findings demonstrate the importance of not just looking at nonreciprocation of tie formation, but also active prohibition of social connection via blocking.

We next ask if these patterns are replicated in Field Experiment 2, in which we added an additional politically neutral control condition. As expected from past work, when predicting whether the user followed-back our bot account, we found that users were significantly less likely to follow back the counter-partisan bot compared to the politically neutral bot (*b* = −0.072, SE = 0.012, *t*(3,994) = −6.013, *P* < 0.001); and that this selective follow-back behavior was similar for Democrats and Republicans (no significant interaction between the counter-partisan dummy and the user partisanship dummy, *b* = 0.004, SE = 0.012, *t*(3,994) = 0.320, *P* = 0.749). We also found that users were about 1.47 times more likely to follow back the copartisan bot compared to the politically neutral bot (*b* = 0.055, SE = 0.012, *t*(3,994) = 4.582, *P* < 0.001).

We next predict whether users blocked our accounts by a copartisan dummy variable, counter-partisan dummy variable, user partisanship, and interactions between the shared partisanship variables and user partisanship. As in Field Experiment 1, we find a strong effect of shared partisanship on the likelihood of users blocking our accounts. Users in our sample were about 4.32 times more likely to block a counter-partisan account compared to a politically neutral account (*b* = 0.068, SE = 0.008, *t*(3,994) = 8.993, *P* < 0.001; see Fig. 2b). We did not find evidence for a difference between blocking rates of copartisan accounts compared to politically neutral accounts (*b* = −0.005, SE = 0.008, *t*(3,994) = −0.696, *P* = 0.487). Further replicating our initial field study, we again find evidence for partisan asymmetry in blocking by shared partisanship: more Democratic users were about 4.4 times more likely to block counter-partisan accounts relative to politically neutral accounts whereas Republican users were about 3.6 times more likely to block counter-partisan accounts relative to the control (interaction coefficient: *b* = −0.042, SE = 0.008, *t*(3,994) = −5.486, *P* < 0.001). Thus, both the selective blocking of counter-partisans, and the asymmetry in which this behavior is more pronounced for Democrats than Republicans, successfully replicated.

### Results from survey experiment replication

We next turn to our survey experiment, in which we aimed to replicate our field results in a different context with a different subject pool, and to also shed light on underlying reasons for choosing to block other users. For instance, prior work has suggested that social media users may block accounts for reasons such as managing potential harm and risk (34) or curating personal news consumption (35). We recruited Twitter users through Lucid (*n* = 606) and showed them one of three different Twitter account profiles (politically neutral, Democrat-favoring, Republican-favoring). To mirror our field experiment, we then asked them to suppose that the account had just followed them. We also varied whether we told participants that the account had also liked several of the participants’ past tweets before following them (as in Ref. (36)). Unlike our field experiments, the Twitter account profiles we showed participants had no information about any content the accounts shared. Participants were then asked to select whether they would follow-back the account, ignore the account, or block the account.

**Fig. 3.**
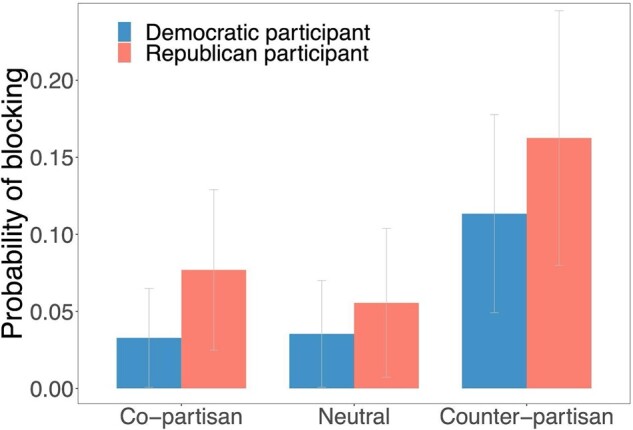
Probability of blocking by shared partisanship and user partisanship in the survey experiment. Error bars indicate 95% CIs.

We used a linear model predicting whether participants selected to block the account, using a copartisanship dummy, a counter-partisanship dummy, participant partisanship (1–7; *z*-scored), whether the account was described as liking tweets before following dummy (centered), and all interactions between shared partisanship, participant partisanship, and liking condition. Replicating our field results, we found that participants were about 3 times more likely to block the counter-partisan profile compared to the politically neutral profile (*b* = 0.089, SE = 0.027, *t*(605) = 3.31, *P* = 0.001; see Fig. 3), and found no significant difference between the copartisan account and the politically neutral account (*b* = 0.006, SE = 0.025, *t*(605) = 0.25, *P* = 0.803). We also replicated these findings in two other supplementary survey experiments with similar designs (see Supplementary material). The increased blocking rate observed for counter-partisans is also notable in comparison to relative follow-back rates—participants were only 1.2 times more likely to follow back the neutral account relative to the counter-partisan account (*b* = −0.088, *P* = 0.080), and only 1.1 times more likely to follow back the copartisan account relative to the neutral account (*b* = 0.056, *P* = 0.238). In contrast to the results of our field experiments, we did not find evidence for significant partisan asymmetries in the blocking of counter-partisans in our survey experiment (no significant interaction between counter-partisan dummy and user partisanship, *b* = 0.023, SE *=* 0.027, *t*(605) = 0.85, *P* = 0.396). However, this interaction between counter-partisanship and user partisanship was not very precisely estimated given our sample size—the 95% CI of our estimate suggests our results are consistent with interactions between −0.030 and 0.075. Nonetheless, we do observe statistically significant evidence that the interaction effect we observe in our comparable field experiment (Field Experiment 2) is significantly more negative than that which we observe in our survey experiment (*z*-test between coefficients: *z* = 2.379, *P* = 0.017). This indicates that we see less of the asymmetry in our survey experiment that we first observed in the field. Democrats are more likely to block Republicans than vice versa in the field—but this asymmetry is significantly smaller in our survey results.

### Blocking based on shared content (vs. profiles)

As described above, one important difference between our survey and field experiments is that the accounts in our survey experiment had no prior posts, whereas our field experiment bot accounts retweeted posts from partisan news organizations and elites prior to following users. This means that our bot accounts differed not only in their explicit partisan identification but also in the type of content they retweeted. For example, our Republican bots retweeting from right-leaning news outlets and elites may have shared lower quality information, given evidence of Republican elites making more false claims (37) and sharing more misinformation (38) than their Democratic counterparts. In a separate survey study probing partisan differences in blocking (*n* = 3,057 recruited from Lucid using nationally representative quotas), we asked participants how likely they would be to mute, block, or unfollow people on social media who share various types of content (see SI Section 4 for full methods and results). We found that more Democratic participants were significantly more likely to say they would block users who share content that is (i) inaccurate or false, (ii) mean or nasty, or (iii) racist, sexist, or contains hate speech (*P* < 0.001). Conversely, we found that more Republican participants were significantly more likely to say they would block users who share content that (i) is “woke” or engages in “cancel culture” or (ii) questions or doubts the existence of God (*P* < 0.001).

We also asked about the likelihood of participants’ blocking others on social media who either criticize one's in-party or praise one's out-party—and we varied whether we asked about party elites (Trump, Biden) or partisans (Republicans, Democrats). Interestingly, we found that participants were more likely to block users who praised their out-party than users who criticized their in-party (*P* < 0.001); and observed that this was particularly true for Democrats (interaction between user politics and criticize vs. praise: *P* < 0.001; for full results, see Table S55 and Fig. S6).

In Supplementary Survey Experiment 2, we also find that Democrats are more likely to block an explicitly toxic, trolling account than Republicans (toxic bio: “Professional Troll | Hate speech is free speech | #FightMe”; see Table S34 and Fig. S4). This provides some further evidence of partisan asymmetries in blocking by the content and quality of posts, beyond blocking based on just partisanship per se.

Altogether, these findings suggest that our observation of a greater partisan asymmetry (such that Democrats block Republicans more than vice versa) in the field than in the survey could potentially be explained by partisan differences in the content that our bot accounts retweeted in the field experiments. To evaluate this possibility, we quantified the quality of content our bots shared based on domain-level quality ratings (38), quantified partisan slant of news domains shared using political leanings of domains (39), and measured the use of toxic language using the Google Perspective API (https://perspectiveapi.com/). Indeed, in both field experiments, we found that the Republican bots shared lower quality (Field Experiment 1: *b* = −0.473, *P* < 0.001; Field Experiment 2: *b* = −0.639, *P* < 0.001), more politically slanted (Field Experiment 1: *b* = 1.787, *P* < 0.001; Field Experiment 2: *b* = 1.492, *P* < 0.001), and more toxic content relative to the Democratic bots (Field Experiment 1: *b* = 0.118, *P* < 0.001; Field Experiment 2: *b* = 0.196, *P* < 0.001; see Table 1; SI Section 5). This supports the conclusion that we observed a greater partisan asymmetry in the field than in our survey experiment, in the direction of Democrats engaging in more preferential blocking than Republicans in the field, because the Republican bots in the field were reposting lower quality and more polarized content.

**Table 1.** Average quality, slant, and toxicity scores of the content retweeted by our human-looking bot accounts in Field Experiments 1 and 2, by partisanship.

Experiment	Bot partisanship	*n* posts	Quality score	Slant score	Toxicity score
Field Experiment 1	Democratic	125	0.50	−0.337	0.043
Field Experiment 1	Republican	111	0.409	0.696	0.049
Field Experiment 2	Democratic	105	0.647	−0.210	0.065
Field Experiment 2	Neutral	88	0.866	−0.099	0.053
Field Experiment 2	Republican	90	0.526	0.613	0.078

For comparative analyses, see SI Section 5.

### Reasons why people block others on social media (survey experiment results)

Our findings in the previous section suggest that one motivation for blocking an account may be that account sharing low-quality content. In this final section, we shed further light on *why* people chose to block accounts in our study. Commensurate with the previous section, one reason for blocking could be to filter out seeing unpleasant, low-quality, or counter-partisan content. Individuals may also chose to block others for interpersonal reasons, such as concern about other users seeing their own content, or to mitigate risk of conflict or being “trolled.”

**Fig. 4.**
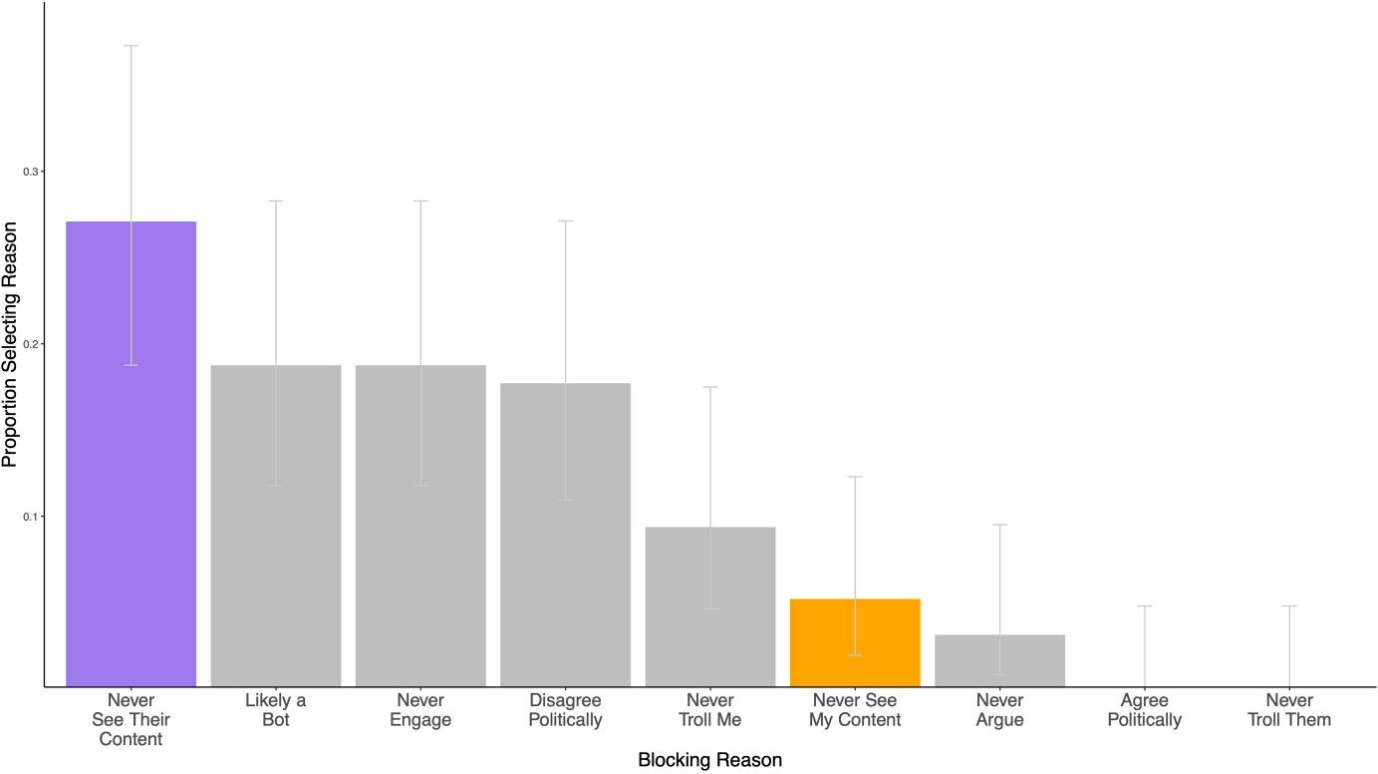
Proportion of participants selecting each reason for blocking the account that followed them, conditional on having blocked the account. Data are combined across Main Survey Experiment and Supplementary Survey Experiment 1 (*n* = 96 participants blocked account in experiments). Error bars reflect 95% CIs.

To differentiate between these possibilities, after participants decided whether or not to block the account in our survey experiment, we asked them a follow-up question on the most important reason why they decided to block the user that followed them. Participants were asked to choose between nine potential options (see Methods). Figure 4 shows the proportion of participants who selected each reason for blocking, conditional on having chosen to block the account that followed them (to increase power, we pool response across both our main survey experiment and a nearly identical Supplementary Survey Experiment 1; across the two experiments, *n* = 96 participants blocked accounts out of 1,240 total participants).

Interestingly, the most commonly selected reason for blocking was participants never wanting to see the content the account tweets and retweets (27%). A one-sample proportion test shows that the proportion of participants selecting this reason for blocking is significantly greater than the proportion blocking because they never want the account to see the content they themselves tweet and retweet (5.2%; χ^2^ = 88.67, *P* < 0.001). This finding provides some evidence that individuals may be using blocking more as a tool for feed content curation, rather than as a means of preventing users from viewing and interacting with their own profile and posts.

## Discussion

Overall, our results shed light on an understudied microfoundation of partisan assortment: preferential social tie *prevention*. While most prior research has examined the causal foundations of political segmentation in terms of social tie formation (i.e. homophilous preferences), our results suggest that the proactive prevention of social connections between counter-partisans is also a powerful causal driver of partisan assortment in social networks. Across two Twitter field experiments, social media users were much more likely to block counter-partisans than copartisans or politically neutral accounts. Furthermore, the magnitude of the effect of (non)shared partisanship is comparable to, or even greater for, tie prevention vs. reciprocation. For example, in our second field experiment, Twitter users were 1.47 times more likely to follow back copartisans than a politically neutral account but were roughly 4 times more likely to block counter-partisans than a politically neutral account. Thus, despite nearly all prior work focusing on associative preferences for establishing social ties with copartisans, selective prevention of ties with counter-partisans appears to be as, or more, important. It is therefore critical for tie prevention to receive more attention in future work, for exampling exploring the psychology underlying, and practical implications of, preferential prevention of ties with counter-partisans.

Our results also add to the current understanding of online partisan dynamics by demonstrating a stark partisan asymmetry in blocking behavior among partisans on Twitter. Across both field experiments, Democratic users were much more likely to block Republican accounts than Republican users were to block Democratic accounts. Interestingly, although our follow-up survey experiment replicated our main result of preferential counter-partisan blocking, we found that Democrats engaged in significantly greater preferential blocking relative to Republicans in the field experiment vs. in our survey experiment. Democrats were more likely to preferentially block than Republicans on Twitter than in our survey. To explain this difference in results, we provide convergent evidence that this may be attributable to differences in the content retweeted by our partisan bot accounts in the field experiments. Additional survey research indicates that Democrats report a greater propensity to block accounts that share inaccurate or false content. While our survey experiment did not provide information about the content shared by our Democratic and Republican profiles, our field experiment accounts retweeted content from right- and left-leaning news outlets—leading to significant differences in the quality, political slant, and toxicity of this retweeted content, such that the content retweeted by our Republican accounts was lower quality, more slanted, and more toxic. As a result, the partisan asymmetry in blocking observed in our field experiments seems likely to have been driven by differences in sharing behavior of the bot accounts, rather than their identities per se.

Theoretically, our results have several key implications. First, the large effects of shared partisanship on blocking behavior—as big or bigger than preferential tie formation—indicate that when studying digital partisan dynamics, blocking and related tie prevention behaviors cannot be ignored. In order to understand the causal foundations of partisan assortment, all potential types of partisan connections should be examined—including not just the formation and dissolution of connections, but also the proactive prevention of cross-partisan ties. For example, future work should use formal models and computer simulations to investigate how preferential tie prevention affects both the structure of online social networks and, more importantly, the flow of information across those networks.

Second, our findings are the first to shed light on the motivations behind blocking behavior on Twitter. We show that the primary reason Twitter users report blocking others is to avoid seeing the content from blocked users—rather than because of political identity per se, or out of concern for blocked users viewing or interacting with one's own posts. This indicates that individuals are commonly using social tie prevention on Twitter due to informational motivations, as a way to curate their newsfeed. This fills an important gap in the literature on why individuals may engage in selective blocking, and this informational curation perspective stands in contrast to more interpersonal motives suggested by affective polarization perspectives. In other words, blocking on Twitter should not be understood as driven by partisan animus alone, nor only by concern about uncivil comments or engagement with counter-partisans (though the latter is still an important concern for user safety online (40)). One potential implication is that attempts to reduce affective polarization may not necessarily impact counter-partisan blocking rates, since blocking is less about punitive action than it is about online content curation. Similarly, decreasing concerns about partisan animus from political adversaries (41) may also reduce affective polarization without altering blocking rates, since users are less concerned with trolling or engagement by counter-partisans than they are with having to view the content posted from counter-partisans.

Given the informational concerns of users who block counter-partisans on Twitter, future research may further investigate whether users preferentially block counter-partisan social vs. purely informational accounts, and whether this varies by the rate of content posting. For instance, users may be more likely to block counter-partisan accounts that frequently tweet or retweet content. Furthermore, our current work does not evaluate the normative or downstream implications of counter-partisan blocking and subsequent online political segmentation. Future work could also examine the effects of selective counter-partisan blocking on important outcomes such as political knowledge, online toxicity, and partisan animosity.

Practically, our results have several important implications for platform design and behavioral intervention delivery. Currently on Twitter (X), blocking is a more difficult procedure than following-back—it requires first navigating to a user's profile and selecting a “more options” button before being able to block, whereas tie formation is achieved via simply clicking a “Follow” button. Nonetheless, our field experiment results suggest that some users still take advantage of the ability to block other users—particularly counter-partisans. Future research could examine the effects of increasing or decreasing the ease of blocking (relative to following-back, unfollowing, or ignoring) on social media, and how this may impact political segmentation and related dynamics of information flow online. Our survey results also show that although many individuals attribute their blocking decisions to not wanting to see the content of blocked users, there are a myriad of possible reasons for blocking users. Thus, social media companies should regularly monitor and ask users why they choose to block accounts—and perhaps consider the development of more and less stringent tie prevention options according to the demands of users. For example, users frequently blocked for toxicity or trolling should perhaps be subject to greater on-platform restrictions than users frequently blocked for repetitive posting.

Another practical implication is that attempts to deliver content or interventions to social media users will likely be more effective if done via politically congenial or neutral profiles, given that counter-partisan accounts are more likely to be blocked. For example, attempts to provision social fact-checks to users who share misinformation may be less effectively delivered by counter-partisan vs. copartisan profiles, since counter-partisan profiles are more likely to be blocked prior to sending a corrective message (36).

Our current work has several limitations to note. First, our bot accounts in our field and survey experiments were portrayed as White, male profiles. Previous field experiments on Twitter have demonstrated important differences in treatment effects of interventions from bots presenting as different races and genders (42, 43). Future research should further investigate how differences in race, gender, and other demographics may moderate preferential blocking and follow-back preferences, as well as their potential moderation of partisan social dynamics. Second, our experiments were performed in the context of Twitter, presenting potential constraints on generality. Our Twitter field experiment examined politically active Twitter users, and our survey experiment only included respondents who reported having Twitter accounts—thus, our samples are not representative of Twitter users, nor Americans in general. Furthermore, the blocking dynamics observed may be specific to Twitter—research on other platforms such as Facebook and LinkedIn may examine whether similar blocking patterns emerge, and whether blocking on those platforms is also primarily motivated by informational concerns, or perhaps could be more punitive or protectionary in nature. Relatedly, our field experiments were conducted on Twitter in 2020 and 2022—although we observed similar results in both experiments, recent changes in Twitter's ownership (accompanied by its rebranding as “X”) may also make these Twitter-based results less generalizable to today's iteration of the platform. Furthermore, the magnitude of counter-partisan blocking may be affected by important offline political events, such as national elections. That said, our results still convincingly show a pattern of preferential counter-partisan blocking across multiple years. Third, the current work does not examine the downstream implications of preferential counter-partisan blocking. While blocking counter-partisans may increase political homophily, it could also foster tangible benefits—for instance, preferentially blocking users more likely to share inaccurate or false information (e.g. conservatives (44)) could increase the quality of consumed news content for counter-partisans. Furthermore, while our current findings suggest that counter-partisan blocking of our political accounts was mostly attributable to not wanting to see the content they post, it could well be the case that other types of profiles are blocked as a means of safety and harassment reduction—blocking provides a tool for mitigation of harassment and abuse from specific other users, beyond its utility as a content moderation mechanism (27).

In sum, we provide evidence that a fundamental yet overlooked causal contributor to online partisan homophily is selective social tie prevention. Partisans selectively block counter-partisans online. Studies of network dynamics and partisan assortment must consider all potential avenues of social tie maintenance—including social tie prevention—in order to more completely understand propensities to connect and share information within and across party lines.

## Methods

### Field Experiment 1

We identified Twitter users who tweeted or retweeted the hashtags #Trump2020 or #VoteBidenHarris2020 on 2020 October 6, retrieved the timeline of these users up to 3,200 recent (re)tweets, and collected lists of accounts they followed and of their followers. We classified the partisanship of users based on the political leaning of the content they shared (33). We excluded users with more than 15,000 followers (since such users are likely to have high status on the platform and thus less likely to engage with our accounts), users with zero friends or zero followers as these accounts may not be active, and users for whom we could not estimate political partisanship. We constructed a politically balanced set of Twitter users as the subject pool of our experiment.

To balance the users’ characteristics across experimental conditions and improve the precision of our causal estimates, we conducted stratified (i.e. blocked) randomization (45). We constructed relatively homogeneous strata of users based on (i) the log-transformed number of followers (we used number of followers as a proxy for the status of the user and log-transformed the data since followers counts are highly skewed), (ii) the number of tweets in the past 14 days (to measure recent activity level on the platform), (iii) the user’s follow-back rate, which was measured by the number of mutual friendships (accounts with whom the user has reciprocal links) divided by the total number of followers (as a proxy for the baseline user interaction with followers), (iv) user partisanship (Republican vs. Democrat), and (v) the extremity of user partisanship (absolute value of continuous estimated partisanship). We then used these strata to randomly assign users to be followed by either copartisan or counter-partisan bot accounts.

Each of our bot accounts had existed for roughly 3 months, randomly tweeted roughly every 3 days (tweets also reflect bots’ political partisanship) and had about 500 initial politically neutral followers before interacting with the users (in order to appear authentic). The initial followers also helped avoid noise introduced by path dependency (i.e. the probability of interaction with the account could be affected by the number of followers, thus we started with a relatively high number of followers for all bots). Bot profile pictures were generated using GANs and did not include pictures of real individuals. We successfully followed *n* = 2,010 users (median follower count of 426, a median following count of 897, and a mean reciprocity rate of 0.53 as defined by the number of reciprocated links divided by the number of users followed) over the course of 10 days (from 2020 December 2 to 2020 December 11).

The analyses reported were not preregistered.

### Field Experiment 2

We identified Twitter users who retweeted content from the left-leaning mainstream news outlet MSNBC and right-leaning mainstream news outlet Fox News in 2022 February 25, retrieved all (re)tweets up to recent 3,200 posts, and collected the lists of accounts they followed of their followers. We classified political partisanship of the users based on political leaning of the content they shared using (33). We removed users who had more than 15,000 followers, those who had followed fewer than 10 accounts (i.e. inactive users), and those for whom we could not estimate political partisanship.

To assign users with similar characteristics across experimental conditions, we used stratified randomization (45). We constructed approximately homogeneous strata of users based on the same five criteria as in Field Experiment 1. We then used these strata to randomly assign users to be followed by either a copartisan, politically neutral, or counter-partisan bot account. Additionally, we randomly assigned strata of users to be followed by our accounts on different days such that we have a similar number of users across experimental conditions each day.

Prior to conducting the experiment, our bot accounts had approximately 250 politically neutral followers and retweeted content from a mainstream outlet aligned with their apparent political identification. Bot profile pictures were generated using GANs and did not include pictures of real individuals. We used our accounts to follow users over 2 months (from 2022 March 9 to 2022 May 10; note that Field Experiment 1 was conducted in December 2020—our similar findings across both field experiments therefore reflect some generalizability over time in preferential counter-partisan blocking behavior). We successfully followed *n* = 2,003 Twitter users (median follower count of 193, a median following count of 432, and a mean reciprocity rate of 0.407).

The analyses reported were not preregistered.

### Field experiment ethics

For both field experiments, we obtained a waiver of informed and approval for our experimental design from the MIT Committee on the Use of Humans as Experimental Subjects (COUHES) protocol #1907910465.

### Survey experiment

As preregistered (https://aspredicted.org/blind.php?x=P6M_TR1), we recruited participants from Lucid who reported having a Twitter account, with adjusted quotas to better reflect a representative US Twitter population sample by age, gender, and ethnicity (46). We recruited 606 participants who passed trivial attention screeners and began the main survey task (mean age = 39.98, 293 female, 412 White-only). Participants first completed basic demographics, political orientation, and Twitter usage questions.

Next, participants were told to suppose they were on Twitter and followed by a given account. Participants were then randomly assigned to one of three account follower conditions: Democrat-favoring (bio: #DefendingOurDemocracy #BidenHarris2014), Republican-favoring (bio: #MAGA #Trump2024), or politically neutral (bio: Product Manager | Amateur chef | #Photographer). Participants were also assigned to either a prior-engagement or control condition—participants in the prior-engagement condition were additionally instructed to suppose that the account that followed them had liked three recent tweets of theirs. Follower and prior liking information was shown via a simulated Twitter “Notifications” screen. The account profile picture was generated using a GAN and did not include a picture of a real individual. Participants were then asked “How would you respond to this notification that Thomas Maddocks [the account name] follows you?” (Follow this user back; Ignore this notification; Block this user). Participants were then asked to write several sentences about why they either followed, ignored, or blocked the user. Then, participants were asked a multiple choice question to select the most important reason why they decided to follow, ignore, or block the user. Users who chose to block the user selected from the following list of possible reasons: (i) “I never want to see the content they tweet and retweet,” (ii) “I never want them to see the content I tweet and retweet,” (iii) “I agree with them politically,” (iv) I disagree with them politically,” (v) “I never want to engage in online discussion with them,” (vi) “I never want to argue with this person,” (vii) “I do not want to troll or “dunk on” this person,” (viii) “I do not want this person to troll or “dunk on” me,” and (ix) “I believe this account is a bot.”

Two additional survey experiments (reported in Supplementary material) followed nearly identical procedures. We also report our partisan differences in blocking grid item procedure in Supplementary material.

### Survey experiment ethics

All survey experiments received exempt evaluations via the MIT COUHES protocol E-4690.

## Supplementary Material

pgae161_Supplementary_Data

## Data Availability

Data, analysis files, and materials are available at https://osf.io/46aqr/.
