# Supplementary material for: Blocking of counter-partisan accounts drives political assortment on Twitter
Source: PNAS Nexus. 2024 Apr 15;3(5):pgae161. doi: 10.1093/pnasnexus/pgae161 (PMC11110939; doi:10.1093/pnasnexus/pgae161)
Supplement: pgae161_Supplementary_Data [file pgae161_supplementary_data.docx]

**Supplementary Information:**

***Blocking of counter-partisan accounts drives political assortment on Twitter***

**Table of Contents.**

1. **Main Survey Experiment.**
2. **Supplementary Survey Experiment 1.**
3. **Supplementary Survey Experiment 2.**
4. **Blocking Partisan Differences - Grid Item.**
5. **Partisan Bot Content in Twitter Field Experiments.**
6. **Field Experiment 1 Analyses.**
7. **Field Experiment 2 Analyses.**
8. **Main Survey Experiment.**

**Open Science Statement.**

We pre-registered our survey experiment here: <https://aspredicted.org/blind.php?x=P6M_TR1>. We report all pre-registered analyses here. Three deviations in reported analyses are (i) we report descriptive statistics for blocking reasons, rather than multiple analyses per multiple choice option, due to data sparsity, (ii) we do not use natural language processing techniques or raters to evaluate the free-response items collected because of data sparsity (though free response data are available on this project’s OSF page), and (iii) instead of blocking knowledge as a moderator, we report analyses filtering by blocking knowledge.

Survey materials, data, and analysis files are available here: <https://osf.io/46aqr/?view_only=3a7f16b62131490aabb83a782fa133e5>.

**Table S1: Demographics.**

| ***N*** | 606 |
| --- | --- |
| **Gender** | 293 Female,  310 Male,  3 Other |
| **Age** | *M*_age_= 39.98 |
| **Race/Ethnicity** | 412 White (only) |

**Table S2: Blocking Predicted by User Partisanship, Account Partisanship, and Engagement.**

|  | ***b*** | ***SE*** | ***t*** | ***p*** |
| --- | --- | --- | --- | --- |
| (Intercept) | 0.046 | 0.018 | 2.514 | 0.012* |
| UserPolitics | 0.005 | 0.019 | 0.257 | 0.797 |
| DemBot | 0.044 | 0.026 | 1.696 | 0.09 |
| RepBot | 0.05 | 0.026 | 1.919 | 0.056 |
| EngageIV | 0.032 | 0.037 | 0.865 | 0.388 |
| UserPolitics:DemBot | 0.059 | 0.026 | 2.247 | 0.025* |
| UserPolitics:RepBot | -0.021 | 0.026 | -0.803 | 0.422 |
| UserPolitics:EngageIV | 0.044 | 0.037 | 1.176 | 0.24 |
| DemBot:EngageIV | -0.081 | 0.052 | -1.557 | 0.12 |
| RepBot:EngageIV | -0.077 | 0.052 | -1.471 | 0.142 |
| UserPolitics:DemBot:EngageIV | -0.061 | 0.052 | -1.172 | 0.242 |
| UserPolitics:RepBot:EngageIV | -0.067 | 0.052 | -1.287 | 0.198 |

**p*<.05, ***p*<.01, ****p*<.001

Note: Linear model predicting blocking decision (1=block, 0=follow or ignore) by user partisanship (1=Strongly Democratic, 7=Strongly Republican; *z*-scored), bot partisanship condition dummies (baseline = neutral control; dummies for Democratic Bot, Republican Bot), and engagement condition (centered; -0.5=no engagement, 0.5=engagement), allowing for all interactions.

**Table S3: Blocking Predicted by Shared Partisanship, User Partisanship, and Engagement.**

|  | ***b*** | ***SE*** | ***t*** | ***p*** |
| --- | --- | --- | --- | --- |
| (Intercept) | 0.046 | 0.018 | 2.516 | 0.012* |
| Concord | 0.006 | 0.025 | 0.249 | 0.803 |
| Discord | 0.089 | 0.027 | 3.309 | 0.001*** |
| UserPolitics | 0.005 | 0.019 | 0.258 | 0.797 |
| EngageIV | 0.032 | 0.037 | 0.865 | 0.387 |
| Concord:UserPolitics | 0.016 | 0.025 | 0.639 | 0.523 |
| Discord:UserPolitics | 0.023 | 0.027 | 0.85 | 0.396 |
| Concord:EngageIV | -0.083 | 0.05 | -1.648 | 0.100 |
| Discord:EngageIV | -0.073 | 0.054 | -1.366 | 0.173 |
| UserPolitics:EngageIV | 0.044 | 0.037 | 1.177 | 0.24 |
| Concord:UserPolitics:EngageIV | -0.076 | 0.051 | -1.501 | 0.134 |
| Discord:UserPolitics:EngageIV | -0.05 | 0.054 | -0.931 | 0.352 |

**p*<.05, ***p*<.01, ****p*<.001

Note: Linear model predicting blocking decision (1=block, 0=follow or ignore) by political concordance dummy, political discordance dummy (concordance and discordance for political preference = 4 determined via follow-up binary forced choice partisanship item), partisanship (*z*-scored), engagement condition (centered), and interactions between shared partisanship dummies, user partisanship, and engagement.

**Table S4: Blocking Counts & Reasons by User-Account Matching.**

| **User - Account** | **Proportion Block** | **Never Want Their Content** | **Never Want Them to See My Content** | **Agree Politically** | **Disagree Politically** | **Never Want To Engage** | **Never Want To Argue** | **Do Not Want to Troll Them** | **Do Not Want to Troll Me** | **Likely A Bot** |
| --- | --- | --- | --- | --- | --- | --- | --- | --- | --- | --- |
| Dem-Rep | 0.113 (11/97) | 2 | 1 | 0 | 3 | 3 | 0 | 0 | 2 | 0 |
| Dem-Dem | 0.033 (4/122) | 3 | 0 | 0 | 0 | 0 | 0 | 0 | 0 | 1 |
| Dem-Neu | 0.035 (4/113) | 2 | 0 | 0 | 0 | 0 | 0 | 0 | 1 | 1 |
| Rep-Rep | 0.077 (8/105) | 2 | 1 | 0 | 0 | 2 | 0 | 0 | 0 | 3 |
| Rep-Dem | 0.163 (13/80) | 3 | 1 | 0 | 4 | 2 | 0 | 0 | 2 | 1 |
| Rep-Neu | 0.056 (5/90) | 1 | 0 | 0 | 0 | 2 | 0 | 0 | 1 | 1 |
| **Total** | — | **13** | **3** | **0** | **7** | **9** | **0** | **0** | **6** | **7** |

**Table S5: Follow-Back Predicted by User Partisanship, Account Partisanship, and Engagement.**

|  | ***b*** | ***SE*** | ***t*** | ***p*** |
| --- | --- | --- | --- | --- |
| (Intercept) | 0.549 | 0.034 | 15.939 | <0.001*** |
| UserPolitics | -0.08 | 0.035 | -2.289 | 0.023* |
| DemBot | -0.042 | 0.049 | -0.868 | 0.386 |
| RepBot | 0.013 | 0.049 | 0.268 | 0.789 |
| EngageIV | 0.043 | 0.069 | 0.629 | 0.53 |
| UserPolitics:DemBot | -0.078 | 0.049 | -1.585 | 0.114 |
| UserPolitics:RepBot | 0.052 | 0.049 | 1.058 | 0.291 |
| UserPolitics:EngageIV | 0.021 | 0.07 | 0.3 | 0.764 |
| DemBot:EngageIV | -0.065 | 0.098 | -0.67 | 0.503 |
| RepBot:EngageIV | 0.111 | 0.098 | 1.133 | 0.258 |
| UserPolitics:DemBot:EngageIV | 0.059 | 0.098 | 0.599 | 0.549 |
| UserPolitics:RepBot:EngageIV | -0.024 | 0.098 | -0.245 | 0.807 |

**p*<.05, ***p*<.01, ****p*<.001

Note: Linear model predicting follow-back decision (1=follow, 0=block or ignore) by user partisanship (1=Strongly Democratic, 7=Strongly Republican; *z*-scored), bot partisanship condition dummies (baseline = neutral control; dummies for Democratic Bot, Republican Bot), and engagement condition (centered; -0.5=no engagement, 0.5=engagement), allowing for all interactions.

**Table S6: Follow-back Predicted by Shared Partisanship, User Partisanship, and Engagement.**

|  | ***b*** | ***SE*** | ***t*** | ***p*** |
| --- | --- | --- | --- | --- |
| (Intercept) | 0.549 | 0.034 | 15.994 | <0.001*** |
| Concord | 0.056 | 0.047 | 1.182 | 0.238 |
| Discord | -0.088 | 0.05 | -1.757 | 0.08 |
| UserPolitics | -0.08 | 0.035 | -2.297 | 0.022* |
| EngageIV | 0.043 | 0.069 | 0.631 | 0.528 |
| Concord:UserPolitics | 0.022 | 0.048 | 0.467 | 0.641 |
| Discord:UserPolitics | -0.052 | 0.05 | -1.035 | 0.301 |
| Concord:EngageIV | -0.031 | 0.094 | -0.332 | 0.74 |
| Discord:EngageIV | 0.078 | 0.1 | 0.779 | 0.437 |
| UserPolitics:EngageIV | 0.021 | 0.07 | 0.301 | 0.764 |
| Concord:UserPolitics:EngageIV | 0.113 | 0.095 | 1.191 | 0.234 |
| Discord:UserPolitics:EngageIV | -0.083 | 0.101 | -0.82 | 0.412 |

**p*<.05, ***p*<.01, ****p*<.001

Note: Linear model predicting follow-back decision (1=follow, 0=block or ignore) by political concordance dummy, political discordance dummy (concordance and discordance for political preference = 4 determined via follow-up binary forced choice partisanship item), partisanship (*z*-scored), engagement condition (centered), and interactions between shared partisanship dummies, user partisanship, and engagement.

**Table S7: Blocking Predicted by User Partisanship, Account Partisanship, and Engagement; Filtering for Attention (*N*=484).**

|  | ***b*** | ***SE*** | ***t*** | ***p*** |
| --- | --- | --- | --- | --- |
| (Intercept) | 0.057 | 0.021 | 2.713 | 0.007** |
| UserPolitics | 0.008 | 0.022 | 0.349 | 0.727 |
| DemBot | 0.057 | 0.03 | 1.874 | 0.062 |
| RepBot | 0.041 | 0.03 | 1.343 | 0.18 |
| EngageIV | 0.042 | 0.042 | 0.989 | 0.323 |
| UserPolitics:DemBot | 0.075 | 0.031 | 2.443 | 0.015* |
| UserPolitics:RepBot | -0.034 | 0.031 | -1.106 | 0.269 |
| UserPolitics:EngageIV | 0.059 | 0.043 | 1.368 | 0.172 |
| DemBot:EngageIV | -0.089 | 0.061 | -1.466 | 0.143 |
| RepBot:EngageIV | -0.099 | 0.06 | -1.632 | 0.103 |
| UserPolitics:DemBot:EngageIV | -0.075 | 0.062 | -1.221 | 0.223 |
| UserPolitics:RepBot:EngageIV | -0.068 | 0.061 | -1.106 | 0.269 |

**p*<.05, ***p*<.01, ****p*<.001

Note: Linear model predicting blocking decision (1=block, 0=follow or ignore) by user partisanship (1=Strongly Democratic, 7=Strongly Republican; *z*-scored), bot partisanship condition dummies (baseline = neutral control; dummies for Democratic Bot, Republican Bot), and engagement condition (centered; -0.5=no engagement, 0.5=engagement), allowing for all interactions; filtering for passing at least 2 of 4 attention check items.

**Table S8: Blocking Predicted by Shared Partisanship, User Partisanship, and Engagement; Filtering for Attention (*N*=484).**

|  | ***b*** | ***SE*** | ***t*** | ***p*** |
| --- | --- | --- | --- | --- |
| (Intercept) | 0.057 | 0.021 | 2.715 | 0.007** |
| Concord | -0.006 | 0.029 | -0.213 | 0.832 |
| Discord | 0.103 | 0.031 | 3.289 | 0.001** |
| UserPolitics | 0.008 | 0.022 | 0.349 | 0.727 |
| EngageIV | 0.042 | 0.042 | 0.989 | 0.323 |
| Concord:UserPolitics | 0.008 | 0.03 | 0.258 | 0.797 |
| Discord:UserPolitics | 0.035 | 0.032 | 1.105 | 0.27 |
| Concord:EngageIV | -0.098 | 0.059 | -1.679 | 0.094 |
| Discord:EngageIV | -0.093 | 0.063 | -1.48 | 0.14 |
| UserPolitics:EngageIV | 0.059 | 0.043 | 1.369 | 0.172 |
| Concord:UserPolitics:EngageIV | -0.082 | 0.059 | -1.375 | 0.17 |
| Discord:UserPolitics:EngageIV | -0.06 | 0.064 | -0.951 | 0.342 |

**p*<.05, ***p*<.01, ****p*<.001

Note: Linear model predicting blocking decision (1=block, 0=follow or ignore) by political concordance dummy, political discordance dummy (concordance and discordance for political preference = 4 determined via follow-up binary forced choice partisanship item), partisanship (*z*-scored), engagement condition (centered), and interactions between shared partisanship dummies, user partisanship, and engagement; filtering for passing at least 2 of 4 attention check items.

**Table S9: Follow-Back Predicted by User Partisanship, Account Partisanship, and Engagement; Filtering for Attention (*N*=484).**

|  | ***b*** | ***SE*** | ***t*** | ***p*** |
| --- | --- | --- | --- | --- |
| (Intercept) | 0.51 | 0.038 | 13.586 | <0.001*** |
| UserPolitics | -0.089 | 0.039 | -2.3 | 0.022* |
| DemBot | -0.044 | 0.054 | -0.802 | 0.423 |
| RepBot | 0.037 | 0.054 | 0.676 | 0.5 |
| EngageIV | 0.04 | 0.075 | 0.53 | 0.596 |
| UserPolitics:DemBot | -0.106 | 0.055 | -1.928 | 0.055 |
| UserPolitics:RepBot | 0.055 | 0.055 | 1.008 | 0.314 |
| UserPolitics:EngageIV | 0.013 | 0.077 | 0.169 | 0.866 |
| DemBot:EngageIV | -0.05 | 0.109 | -0.458 | 0.648 |
| RepBot:EngageIV | 0.145 | 0.108 | 1.34 | 0.181 |
| UserPolitics:DemBot:EngageIV | 0.038 | 0.11 | 0.347 | 0.728 |
| UserPolitics:RepBot:EngageIV | 0.078 | 0.11 | 0.714 | 0.476 |

**p*<.05, ***p*<.01, ****p*<.001

Note: Linear model predicting follow-back decision (1=follow, 0=block or ignore) by user partisanship (1=Strongly Democratic, 7=Strongly Republican; *z*-scored), bot partisanship condition dummies (baseline = neutral control; dummies for Democratic Bot, Republican Bot), and engagement condition (centered; -0.5=no engagement, 0.5=engagement), allowing for all interactions; filtering for passing at least 2 of 4 attention check items.

**Table S10: Follow-back Predicted by Shared Partisanship, User Partisanship, and Engagement; Filtering for Attention (*N*=484).**

|  | ***b*** | ***SE*** | ***t*** | ***p*** |
| --- | --- | --- | --- | --- |
| (Intercept) | 0.51 | 0.037 | 13.673 | <0.001*** |
| Concord | 0.092 | 0.052 | 1.767 | 0.078 |
| Discord | -0.10 | 0.056 | -1.799 | 0.073 |
| UserPolitics | -0.089 | 0.038 | -2.315 | 0.021* |
| EngageIV | 0.04 | 0.075 | 0.534 | 0.594 |
| Concord:UserPolitics | 0.021 | 0.053 | 0.389 | 0.698 |
| Discord:UserPolitics | -0.077 | 0.057 | -1.359 | 0.175 |
| Concord:EngageIV | 0.068 | 0.104 | 0.656 | 0.512 |
| Discord:EngageIV | 0.035 | 0.111 | 0.319 | 0.75 |
| UserPolitics:EngageIV | 0.013 | 0.077 | 0.17 | 0.865 |
| Concord:UserPolitics:EngageIV | 0.171 | 0.106 | 1.623 | 0.105 |
| Discord:UserPolitics:EngageIV | -0.061 | 0.113 | -0.541 | 0.589 |

**p*<.05, ***p*<.01, ****p*<.001

Note: Linear model predicting follow-back decision (1=follow, 0=block or ignore) by political concordance dummy, political discordance dummy (concordance and discordance for political preference = 4 determined via follow-up binary forced choice partisanship item), partisanship (*z*-scored), engagement condition (centered), and interactions between shared partisanship dummies, user partisanship, and engagement; filtering for passing at least 2 of 4 attention check items.

**Table S11: Blocking Predicted by User Partisanship, Account Partisanship, Engagement, and Twitter Usage.**

|  | ***b*** | ***SE*** | ***t*** | ***p*** |
| --- | --- | --- | --- | --- |
| (Intercept) | 0.044 | 0.018 | 2.366 | 0.018* |
| UserPolitics | 0.004 | 0.019 | 0.217 | 0.829 |
| DemBot | 0.044 | 0.026 | 1.69 | 0.092 |
| RepBot | 0.052 | 0.027 | 1.96 | 0.051 |
| EngageIV | 0.028 | 0.037 | 0.748 | 0.455 |
| TwitPC | -0.011 | 0.014 | -0.806 | 0.421 |
| UserPolitics:DemBot | 0.052 | 0.026 | 1.962 | 0.05 |
| UserPolitics:RepBot | -0.029 | 0.027 | -1.092 | 0.275 |
| UserPolitics:EngageIV | 0.045 | 0.037 | 1.197 | 0.232 |
| DemBot:EngageIV | -0.091 | 0.053 | -1.724 | 0.085 |
| RepBot:EngageIV | -0.07 | 0.053 | -1.325 | 0.186 |
| UserPolitics:TwitPC | -0.008 | 0.015 | -0.54 | 0.589 |
| DemBot:TwitPC | -0.013 | 0.02 | -0.678 | 0.498 |
| RepBot:TwitPC | -0.032 | 0.021 | -1.497 | 0.135 |
| EngageIV:TwitPC | 0.006 | 0.028 | 0.196 | 0.845 |
| UserPolitics:DemBot:EngageIV | -0.066 | 0.053 | -1.257 | 0.209 |
| UserPolitics:RepBot:EngageIV | -0.078 | 0.053 | -1.457 | 0.146 |
| UserPolitics:DemBot:TwitPC | -0.001 | 0.02 | -0.026 | 0.98 |
| UserPolitics:RepBot:TwitPC | -0.002 | 0.022 | -0.106 | 0.916 |
| UserPolitics:EngageIV:TwitPC | -0.028 | 0.03 | -0.957 | 0.339 |
| DemBot:EngageIV:TwitPC | -0.04 | 0.04 | -1.003 | 0.316 |
| RepBot:EngageIV:TwitPC | 0.006 | 0.042 | 0.135 | 0.893 |
| UserPolitics:DemBot:EngageIV:TwitPC | -0.003 | 0.039 | -0.077 | 0.939 |
| UserPolitics:RepBot:EngageIV:TwitPC | 0.057 | 0.043 | 1.327 | 0.185 |

**p*<.05, ***p*<.01, ****p*<.001

Note: Linear model predicting blocking decision (1=block, 0=follow or ignore) by user partisanship (1=Strongly Democratic, 7=Strongly Republican; *z*-scored), bot partisanship condition dummies (baseline = neutral control; dummies for Democratic Bot, Republican Bot), engagement condition (centered; -0.5=no engagement, 0.5=engagement), and the first principal component of five Twitter usage covariates (Twitter usage frequency, Twitter tweeting frequency, total number of Tweets, number of accounts followed, number of followers; reverse-coded); allowing for all interactions.

**Table S12: Twitter UsagePCA First Component Loadings.**

|  | PC1 |
| --- | --- |
| Twitter_Frequency | -0.629 |
| Tweeting_Frequency | -0.641 |
| Total_Tweets | -0.009 |
| Total_Following | -0.314 |
| Total_Followers | -0.307 |

**Table S13: Blocking Predicted by Shared Partisanship, User Partisanship, Engagement, and Twitter Usage.**

|  | ***b*** | ***SE*** | ***t*** | ***p*** |
| --- | --- | --- | --- | --- |
| (Intercept) | 0.044 | 0.018 | 2.372 | 0.018* |
| Concord | 0.008 | 0.026 | 0.301 | 0.764 |
| Discord | 0.089 | 0.027 | 3.295 | 0.001** |
| UserPolitics | 0.004 | 0.019 | 0.217 | 0.828 |
| EngageIV | 0.028 | 0.037 | 0.75 | 0.454 |
| TwitPC | -0.011 | 0.014 | -0.808 | 0.419 |
| Concord:UserPolitics | 0.01 | 0.026 | 0.398 | 0.69 |
| Discord:UserPolitics | 0.015 | 0.027 | 0.569 | 0.57 |
| Concord:EngageIV | -0.086 | 0.051 | -1.678 | 0.094 |
| Discord:EngageIV | -0.077 | 0.054 | -1.42 | 0.156 |
| UserPolitics:EngageIV | 0.045 | 0.037 | 1.2 | 0.231 |
| Concord:TwitPC | -0.029 | 0.02 | -1.449 | 0.148 |
| Discord:TwitPC | -0.015 | 0.021 | -0.732 | 0.464 |
| UserPolitics:TwitPC | -0.008 | 0.015 | -0.542 | 0.588 |
| EngageIV:TwitPC | 0.006 | 0.028 | 0.196 | 0.845 |
| Concord:UserPolitics:EngageIV | -0.076 | 0.051 | -1.47 | 0.142 |
| Discord:UserPolitics:EngageIV | -0.065 | 0.054 | -1.202 | 0.23 |
| Concord:UserPolitics:TwitPC | -0.008 | 0.02 | -0.408 | 0.684 |
| Discord:UserPolitics:TwitPC | 0.001 | 0.021 | 0.057 | 0.955 |
| Concord:EngageIV:TwitPC | 0.021 | 0.04 | 0.529 | 0.597 |
| Discord:EngageIV:TwitPC | -0.057 | 0.042 | -1.363 | 0.173 |
| UserPolitics:EngageIV:TwitPC | -0.028 | 0.03 | -0.959 | 0.338 |
| Concord:UserPolitics:EngageIV:TwitPC | 0.06 | 0.04 | 1.476 | 0.14 |
| Discord:UserPolitics:EngageIV:TwitPC | -0.001 | 0.042 | -0.019 | 0.985 |

**p*<.05, ***p*<.01, ****p*<.001

Note: Linear model predicting blocking decision (1=block, 0=follow or ignore) by political concordance dummy, political discordance dummy (concordance and discordance for political preference = 4 determined via follow-up binary forced choice partisanship item), partisanship (*z*-scored), engagement condition (centered), and the first principal component of five Twitter usage covariates (Twitter usage frequency, Twitter tweeting frequency, total number of Tweets, number of accounts followed, number of followers; reverse-coded); allowing for all interactions.

**Table S14: Blocking Predicted by User Partisanship, Account Partisanship, and Engagement; Filtering for Blocking Knowledge (*N*=265).**

|  | ***b*** | ***SE*** | ***t*** | ***p*** |
| --- | --- | --- | --- | --- |
| (Intercept) | 0.068 | 0.028 | 2.439 | 0.015* |
| UserPolitics | 0.006 | 0.029 | 0.205 | 0.838 |
| DemBot | 0.024 | 0.042 | 0.566 | 0.572 |
| RepBot | 0.047 | 0.041 | 1.148 | 0.252 |
| EngageIV | 0.064 | 0.055 | 1.162 | 0.246 |
| UserPolitics:DemBot | 0.063 | 0.045 | 1.412 | 0.159 |
| UserPolitics:RepBot | -0.047 | 0.041 | -1.148 | 0.252 |
| UserPolitics:EngageIV | 0.088 | 0.058 | 1.528 | 0.128 |
| DemBot:EngageIV | -0.18 | 0.084 | -2.148 | 0.033* |
| RepBot:EngageIV | -0.013 | 0.082 | -0.159 | 0.874 |
| UserPolitics:DemBot:EngageIV | -0.172 | 0.09 | -1.914 | 0.057 |
| UserPolitics:RepBot:EngageIV | -0.13 | 0.083 | -1.573 | 0.117 |

**p*<.05, ***p*<.01, ****p*<.001

Note: Linear model predicting blocking decision (1=block, 0=follow or ignore) by user partisanship (1=Strongly Democratic, 7=Strongly Republican; *z*-scored), bot partisanship condition dummies (baseline = neutral control; dummies for Democratic Bot, Republican Bot), and engagement condition (centered; -0.5=no engagement, 0.5=engagement), allowing for all interactions; filtering for correctly answering a multiple choice question on how to block people on Twitter.

**Table S15: Blocking Predicted by Shared Partisanship, User Partisanship, and Engagement; Filtering for Blocking Knowledge (*N*=265).**

|  | ***b*** | ***SE*** | ***t*** | ***p*** |
| --- | --- | --- | --- | --- |
| (Intercept) | 0.068 | 0.028 | 2.446 | 0.015* |
| Concord | -0.027 | 0.04 | -0.677 | 0.499 |
| Discord | 0.097 | 0.043 | 2.257 | 0.025* |
| UserPolitics | 0.006 | 0.029 | 0.206 | 0.837 |
| EngageIV | 0.064 | 0.055 | 1.166 | 0.245 |
| Concord:UserPolitics | 0.014 | 0.04 | 0.335 | 0.738 |
| Discord:UserPolitics | -0.005 | 0.045 | -0.1 | 0.92 |
| Concord:EngageIV | -0.105 | 0.079 | -1.319 | 0.188 |
| Discord:EngageIV | -0.087 | 0.086 | -1.02 | 0.309 |
| UserPolitics:EngageIV | 0.088 | 0.058 | 1.533 | 0.127 |
| Concord:UserPolitics:EngageIV | -0.07 | 0.081 | -0.867 | 0.387 |
| Discord:UserPolitics:EngageIV | -0.237 | 0.091 | -2.602 | 0.01** |

**p*<.05, ***p*<.01, ****p*<.001

Note: Linear model predicting blocking decision (1=block, 0=follow or ignore) by political concordance dummy, political discordance dummy (concordance and discordance for political preference = 4 determined via follow-up binary forced choice partisanship item), partisanship (*z*-scored), engagement condition (centered), and interactions between shared partisanship dummies, user partisanship, and engagement; filtering for correctly answering a multiple choice question on how to block people on Twitter.

**Table S16: Blocking Predicted by User Partisanship, Account Partisanship, and Engagement; Filtering for Past RTing of MSNBC or Fox News (*N*=252).**

|  | ***b*** | ***SE*** | ***t*** | ***p*** |
| --- | --- | --- | --- | --- |
| (Intercept) | 0.024 | 0.023 | 1.036 | 0.301 |
| UserPolitics | -0.005 | 0.022 | -0.252 | 0.801 |
| DemBot | 0.04 | 0.033 | 1.242 | 0.215 |
| RepBot | 0.035 | 0.032 | 1.09 | 0.277 |
| EngageIV | 0.047 | 0.046 | 1.036 | 0.301 |
| UserPolitics:DemBot | 0.062 | 0.03 | 2.037 | 0.043* |
| UserPolitics:RepBot | -0.001 | 0.03 | -0.038 | 0.97 |
| UserPolitics:EngageIV | -0.011 | 0.044 | -0.252 | 0.801 |
| DemBot:EngageIV | -0.176 | 0.065 | -2.695 | 0.008** |
| RepBot:EngageIV | -0.032 | 0.064 | -0.491 | 0.624 |
| UserPolitics:DemBot:EngageIV | -0.102 | 0.061 | -1.676 | 0.095 |
| UserPolitics:RepBot:EngageIV | 0.012 | 0.061 | 0.203 | 0.839 |

**p*<.05, ***p*<.01, ****p*<.001

Note: Linear model predicting blocking decision (1=block, 0=follow or ignore) by user partisanship (1=Strongly Democratic, 7=Strongly Republican; *z*-scored), bot partisanship condition dummies (baseline = neutral control; dummies for Democratic Bot, Republican Bot), and engagement condition (centered; -0.5=no engagement, 0.5=engagement), allowing for all interactions; filtering for self-reported past retweeting of MSNBC or Fox News.

**Table S15: Blocking Predicted by Shared Partisanship, User Partisanship, and Engagement; Filtering for Past RTing of MSNBC or Fox News (*N*=252).**

|  | ***b*** | ***SE*** | ***t*** | ***p*** |
| --- | --- | --- | --- | --- |
| (Intercept) | 0.024 | 0.023 | 1.036 | 0.301 |
| Concord | -0.001 | 0.032 | -0.046 | 0.964 |
| Discord | 0.077 | 0.033 | 2.361 | 0.019* |
| UserPolitics | -0.005 | 0.022 | -0.252 | 0.801 |
| EngageIV | 0.047 | 0.046 | 1.036 | 0.301 |
| Concord:UserPolitics | 0.03 | 0.03 | 0.991 | 0.323 |
| Discord:UserPolitics | 0.032 | 0.031 | 1.027 | 0.305 |
| Concord:EngageIV | -0.048 | 0.064 | -0.758 | 0.449 |
| Discord:EngageIV | -0.158 | 0.065 | -2.415 | 0.017* |
| UserPolitics:EngageIV | -0.011 | 0.044 | -0.252 | 0.801 |
| Concord:UserPolitics:EngageIV | 0.018 | 0.06 | 0.306 | 0.76 |
| Discord:UserPolitics:EngageIV | -0.108 | 0.061 | -1.751 | 0.081 |

**p*<.05, ***p*<.01, ****p*<.001

Note: Linear model predicting blocking decision (1=block, 0=follow or ignore) by political concordance dummy, political discordance dummy (concordance and discordance for political preference = 4 determined via follow-up binary forced choice partisanship item), partisanship (*z*-scored), engagement condition (centered), and interactions between shared partisanship dummies, user partisanship, and engagement; filtering for self-reported past retweeting of MSNBC or Fox News.


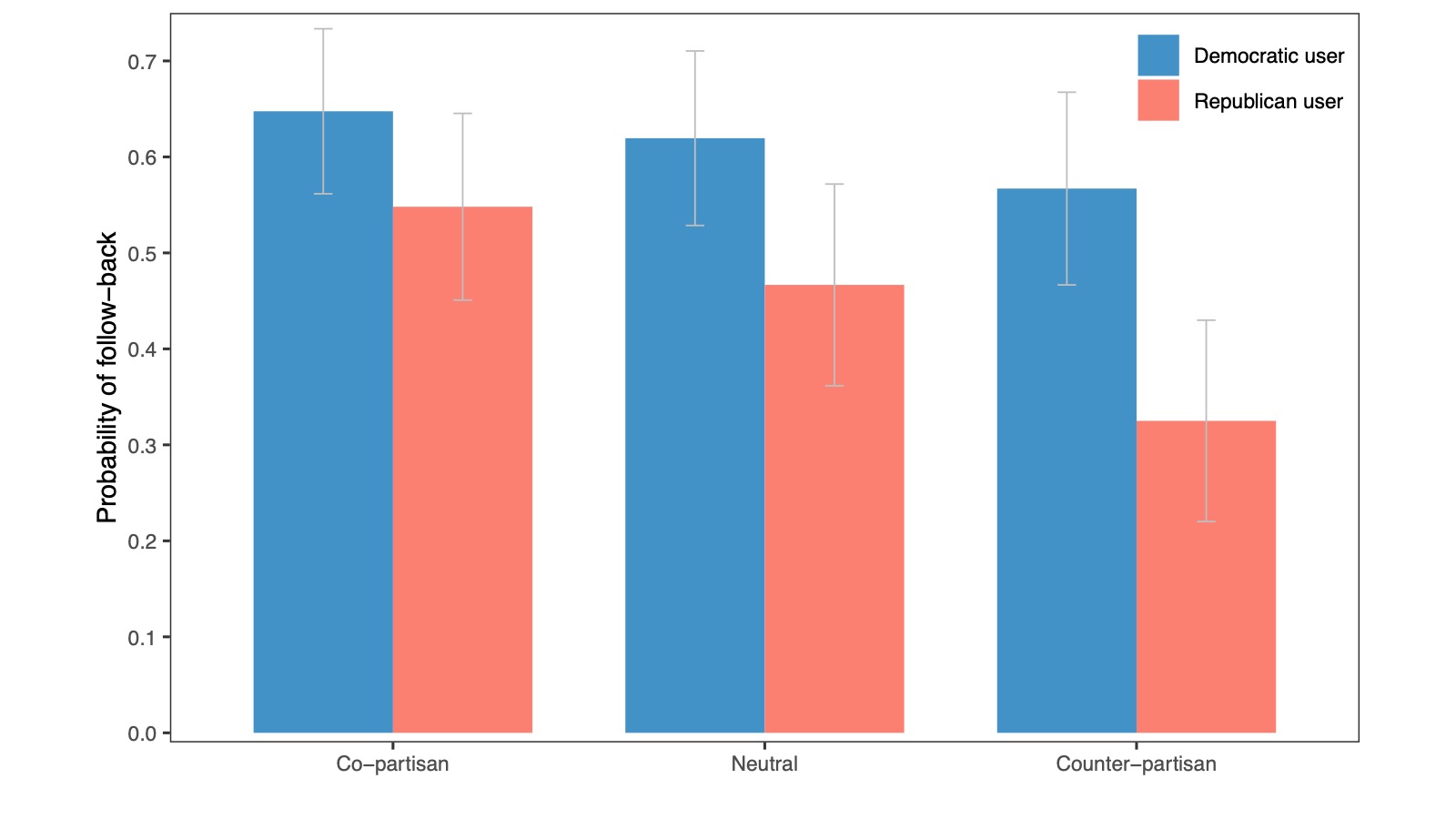


***Figure S1.*** *Probability of follow-back by shared partisanship and user partisanship in survey experiment. Error bars indicate 95% confidence intervals.*

1. **Supplementary Survey Experiment 1.**

**Open Science Statement.**

Supplementary survey experiment 1 followed the same procedure as our main Survey Experiment, and used the same pre-registration: <https://aspredicted.org/blind.php?x=P6M_TR1>. We report all pre-registered analyses here, with the same pre-registration deviations as in our main Survey Experiment.

Survey materials, data, and analysis files are available here: <https://osf.io/46aqr/?view_only=3a7f16b62131490aabb83a782fa133e5>.

**Table S16: Demographics.**

| ***N*** | 634 |
| --- | --- |
| **Gender** | 325 Female,  300 Male,  9 Other |
| **Age** | *M*_age_= 39.86 |
| **Race/Ethnicity** | 430 White (only) |

**Table S17: Blocking Predicted by User Partisanship, Account Partisanship, and Engagement.**

|  | ***b*** | ***SE*** | ***t*** | ***p*** |
| --- | --- | --- | --- | --- |
| (Intercept) | 0.043 | 0.018 | 2.33 | 0.02* |
| UserPolitics | 0.027 | 0.019 | 1.37 | 0.171 |
| DemBot | 0.01 | 0.026 | 0.371 | 0.711 |
| RepBot | 0.103 | 0.026 | 3.928 | <0.001*** |
| EngageIV | -0.028 | 0.037 | -0.751 | 0.453 |
| UserPolitics:DemBot | -0.038 | 0.026 | -1.48 | 0.139 |
| UserPolitics:RepBot | -0.084 | 0.028 | -3.048 | 0.002** |
| UserPolitics:EngageIV | -0.027 | 0.039 | -0.702 | 0.483 |
| DemBot:EngageIV | 0.006 | 0.052 | 0.111 | 0.912 |
| RepBot:EngageIV | -0.011 | 0.052 | -0.208 | 0.835 |
| UserPolitics:DemBot:EngageIV | 0.028 | 0.052 | 0.541 | 0.589 |
| UserPolitics:RepBot:EngageIV | 0.057 | 0.055 | 1.039 | 0.299 |

**p*<.05, ***p*<.01, ****p*<.001

Note: Linear model predicting blocking decision (1=block, 0=follow or ignore) by user partisanship (1=Strongly Democratic, 7=Strongly Republican; *z*-scored), bot partisanship condition dummies (baseline = neutral control; dummies for Democratic Bot, Republican Bot), and engagement condition (centered; -0.5=no engagement, 0.5=engagement), allowing for all interactions.

**Table S18: Blocking Predicted by Shared Partisanship, User Partisanship, and Engagement.**

|  | ***b*** | ***SE*** | ***t*** | ***p*** |
| --- | --- | --- | --- | --- |
| (Intercept) | 0.043 | 0.018 | 2.333 | 0.02* |
| Concord | 0.006 | 0.027 | 0.214 | 0.83 |
| Discord | 0.105 | 0.026 | 4.084 | <0.001*** |
| UserPolitics | 0.027 | 0.019 | 1.371 | 0.171 |
| EngageIV | -0.028 | 0.037 | -0.752 | 0.452 |
| Concord:UserPolitics | -0.04 | 0.028 | -1.461 | 0.144 |
| Discord:UserPolitics | -0.086 | 0.026 | -3.299 | 0.001** |
| Concord:EngageIV | -0.001 | 0.053 | -0.012 | 0.99 |
| Discord:EngageIV | -0.013 | 0.051 | -0.258 | 0.797 |
| UserPolitics:EngageIV | -0.027 | 0.039 | -0.702 | 0.483 |
| Concord:UserPolitics:EngageIV | 0.023 | 0.055 | 0.417 | 0.677 |
| Discord:UserPolitics:EngageIV | 0.055 | 0.052 | 1.05 | 0.294 |

**p*<.05, ***p*<.01, ****p*<.001

Note: Linear model predicting blocking decision (1=block, 0=follow or ignore) by political concordance dummy, political discordance dummy (concordance and discordance for political preference = 4 determined via follow-up binary forced choice partisanship item), partisanship (*z*-scored), engagement condition (centered), and interactions between shared partisanship dummies, user partisanship, and engagement.

**Table S19: Blocking Counts & Reasons by User-Account Matching.**

| **User - Account** | **Proportion Block** | **Never Want Their Content** | **Never Want Them to See My Content** | **Agree Politically** | **Disagree Politically** | **Never Want To Engage** | **Never Want To Argue** | **Do Not Want to Troll Them** | **Do Not Want to Troll Me** | **Likely A Bot** |
| --- | --- | --- | --- | --- | --- | --- | --- | --- | --- | --- |
| Dem-Rep | 0.199 (29/146) | 9 | 1 | 0 | 9 | 5 | 1 | 0 | 1 | 3 |
| Dem-Dem | 0.061 (8/132) | 0 | 0 | 0 | 1 | 2 | 2 | 0 | 1 | 2 |
| Dem-Neu | 0.02 (3/150) | 1 | 0 | 0 | 0 | 0 | 0 | 0 | 0 | 2 |
| Rep-Rep | 0.031 (2/64) | 0 | 0 | 0 | 0 | 0 | 0 | 0 | 0 | 2 |
| Rep-Dem | 0.038 (3/80) | 1 | 0 | 0 | 0 | 1 | 0 | 0 | 0 | 1 |
| Rep-Neu | 0.097 (6/62) | 2 | 1 | 0 | 0 | 1 | 0 | 0 | 1 | 1 |
| **Total** | — | **13** | **2** | **0** | **10** | **9** | **3** | **0** | **3** | **11** |

**Table S20: Follow-Back Predicted by User Partisanship, Account Partisanship, and Engagement.**

|  | ***b*** | ***SE*** | ***t*** | ***p*** |
| --- | --- | --- | --- | --- |
| (Intercept) | 0.442 | 0.034 | 13.124 | <0.001*** |
| UserPolitics | -0.016 | 0.036 | -0.442 | 0.658 |
| DemBot | -0.03 | 0.048 | -0.634 | 0.526 |
| RepBot | -0.066 | 0.048 | -1.373 | 0.17 |
| EngageIV | 0.01 | 0.067 | 0.142 | 0.888 |
| UserPolitics:DemBot | -0.082 | 0.047 | -1.728 | 0.085 |
| UserPolitics:RepBot | -0.082 | 0.05 | -1.621 | 0.106 |
| UserPolitics:EngageIV | -0.014 | 0.071 | -0.2 | 0.841 |
| DemBot:EngageIV | 0.126 | 0.096 | 1.314 | 0.189 |
| RepBot:EngageIV | -0.081 | 0.096 | -0.851 | 0.395 |
| UserPolitics:DemBot:EngageIV | 0.031 | 0.095 | 0.332 | 0.74 |
| UserPolitics:RepBot:EngageIV | -0.062 | 0.101 | -0.619 | 0.536 |

**p*<.05, ***p*<.01, ****p*<.001

Note: Linear model predicting follow-back decision (1=follow, 0=block or ignore) by user partisanship (1=Strongly Democratic, 7=Strongly Republican; *z*-scored), bot partisanship condition dummies (baseline = neutral control; dummies for Democratic Bot, Republican Bot), and engagement condition (centered; -0.5=no engagement, 0.5=engagement), allowing for all interactions.

**Table S21: Follow-back Predicted by Shared Partisanship, User Partisanship, and Engagement.**

|  | ***b*** | ***SE*** | ***t*** | ***p*** |
| --- | --- | --- | --- | --- |
| (Intercept) | 0.442 | 0.034 | 13.179 | <0.001*** |
| Concord | -0.032 | 0.048 | -0.654 | 0.514 |
| Discord | -0.062 | 0.047 | -1.335 | 0.182 |
| UserPolitics | -0.016 | 0.035 | -0.444 | 0.657 |
| EngageIV | 0.01 | 0.067 | 0.142 | 0.887 |
| Concord:UserPolitics | -0.112 | 0.05 | -2.229 | 0.026* |
| Discord:UserPolitics | -0.063 | 0.047 | -1.34 | 0.181 |
| Concord:EngageIV | -0.003 | 0.097 | -0.031 | 0.976 |
| Discord:EngageIV | 0.039 | 0.093 | 0.421 | 0.674 |
| UserPolitics:EngageIV | -0.014 | 0.071 | -0.201 | 0.841 |
| Concord:UserPolitics:EngageIV | -0.174 | 0.1 | -1.735 | 0.083 |
| Discord:UserPolitics:EngageIV | 0.123 | 0.095 | 1.306 | 0.192 |

**p*<.05, ***p*<.01, ****p*<.001

Note: Linear model predicting follow-back decision (1=follow, 0=block or ignore) by political concordance dummy, political discordance dummy (concordance and discordance for political preference = 4 determined via follow-up binary forced choice partisanship item), partisanship (*z*-scored), engagement condition (centered), and interactions between shared partisanship dummies, user partisanship, and engagement.

**Table S22: Blocking Predicted by User Partisanship, Account Partisanship, and Engagement; Filtering for Attention (*N*=503).**

|  | ***b*** | ***SE*** | ***t*** | ***p*** |
| --- | --- | --- | --- | --- |
| (Intercept) | 0.046 | 0.021 | 2.173 | 0.03* |
| UserPolitics | 0.037 | 0.022 | 1.685 | 0.093 |
| DemBot | -0.005 | 0.03 | -0.153 | 0.878 |
| RepBot | 0.125 | 0.03 | 4.163 | <0.001*** |
| EngageIV | -0.043 | 0.043 | -1.004 | 0.316 |
| UserPolitics:DemBot | -0.044 | 0.03 | -1.475 | 0.141 |
| UserPolitics:RepBot | -0.116 | 0.032 | -3.644 | <0.001*** |
| UserPolitics:EngageIV | -0.019 | 0.044 | -0.418 | 0.676 |
| DemBot:EngageIV | 0.012 | 0.06 | 0.197 | 0.844 |
| RepBot:EngageIV | -0.005 | 0.06 | -0.088 | 0.93 |
| UserPolitics:DemBot:EngageIV | 0.018 | 0.059 | 0.304 | 0.762 |
| UserPolitics:RepBot:EngageIV | 0.039 | 0.064 | 0.608 | 0.544 |

**p*<.05, ***p*<.01, ****p*<.001

Note: Linear model predicting blocking decision (1=block, 0=follow or ignore) by user partisanship (1=Strongly Democratic, 7=Strongly Republican; *z*-scored), bot partisanship condition dummies (baseline = neutral control; dummies for Democratic Bot, Republican Bot), and engagement condition (centered; -0.5=no engagement, 0.5=engagement), allowing for all interactions; filtering for passing at least 2 of 4 attention check items.

**Table S23: Blocking Predicted by Shared Partisanship, User Partisanship, and Engagement; Filtering for Attention (*N*=503).**

|  | ***b*** | ***SE*** | ***t*** | ***p*** |
| --- | --- | --- | --- | --- |
| (Intercept) | 0.046 | 0.021 | 2.181 | 0.03* |
| Concord | -0.01 | 0.03 | -0.329 | 0.742 |
| Discord | 0.131 | 0.03 | 4.437 | <0.001*** |
| UserPolitics | 0.037 | 0.022 | 1.691 | 0.092 |
| EngageIV | -0.043 | 0.042 | -1.007 | 0.314 |
| Concord:UserPolitics | -0.043 | 0.032 | -1.353 | 0.177 |
| Discord:UserPolitics | -0.117 | 0.03 | -3.924 | <0.001*** |
| Concord:EngageIV | -0.001 | 0.061 | -0.019 | 0.985 |
| Discord:EngageIV | -0.003 | 0.059 | -0.05 | 0.96 |
| UserPolitics:EngageIV | -0.019 | 0.044 | -0.419 | 0.675 |
| Concord:UserPolitics:EngageIV | 0.011 | 0.063 | 0.166 | 0.868 |
| Discord:UserPolitics:EngageIV | 0.042 | 0.059 | 0.704 | 0.482 |

**p*<.05, ***p*<.01, ****p*<.001

Note: Linear model predicting blocking decision (1=block, 0=follow or ignore) by political concordance dummy, political discordance dummy (concordance and discordance for political preference = 4 determined via follow-up binary forced choice partisanship item), partisanship (*z*-scored), engagement condition (centered), and interactions between shared partisanship dummies, user partisanship, and engagement; filtering for passing at least 2 of 4 attention check items.

**Table S24: Follow-Back Predicted by User Partisanship, Account Partisanship, and Engagement; Filtering for Attention (*N*=503).**

|  | ***b*** | ***SE*** | ***t*** | ***p*** |
| --- | --- | --- | --- | --- |
| (Intercept) | 0.444 | 0.037 | 11.862 | <0.001*** |
| UserPolitics | 0.008 | 0.039 | 0.214 | 0.83 |
| DemBot | -0.04 | 0.053 | -0.752 | 0.452 |
| RepBot | -0.119 | 0.053 | -2.251 | 0.025* |
| EngageIV | 0.05 | 0.075 | 0.668 | 0.505 |
| UserPolitics:DemBot | -0.103 | 0.052 | -1.96 | 0.051 |
| UserPolitics:RepBot | -0.088 | 0.056 | -1.561 | 0.119 |
| UserPolitics:EngageIV | 0.045 | 0.078 | 0.57 | 0.569 |
| DemBot:EngageIV | 0.073 | 0.106 | 0.687 | 0.492 |
| RepBot:EngageIV | -0.126 | 0.106 | -1.192 | 0.234 |
| UserPolitics:DemBot:EngageIV | -0.054 | 0.105 | -0.516 | 0.606 |
| UserPolitics:RepBot:EngageIV | -0.091 | 0.113 | -0.81 | 0.418 |

**p*<.05, ***p*<.01, ****p*<.001

Note: Linear model predicting follow-back decision (1=follow, 0=block or ignore) by user partisanship (1=Strongly Democratic, 7=Strongly Republican; *z*-scored), bot partisanship condition dummies (baseline = neutral control; dummies for Democratic Bot, Republican Bot), and engagement condition (centered; -0.5=no engagement, 0.5=engagement), allowing for all interactions; filtering for passing at least 2 of 4 attention check items.

**Table S25: Follow-back Predicted by Shared Partisanship, User Partisanship, and Engagement; Filtering for Attention (*N*=503).**

|  | ***b*** | ***SE*** | ***t*** | ***p*** |
| --- | --- | --- | --- | --- |
| (Intercept) | 0.444 | 0.037 | 11.906 | <0.001*** |
| Concord | -0.047 | 0.054 | -0.882 | 0.378 |
| Discord | -0.109 | 0.052 | -2.086 | 0.038* |
| UserPolitics | 0.008 | 0.039 | 0.215 | 0.83 |
| EngageIV | 0.05 | 0.075 | 0.67 | 0.503 |
| Concord:UserPolitics | -0.143 | 0.056 | -2.559 | 0.011* |
| Discord:UserPolitics | -0.057 | 0.052 | -1.09 | 0.276 |
| Concord:EngageIV | 0.008 | 0.107 | 0.073 | 0.942 |
| Discord:EngageIV | -0.061 | 0.104 | -0.589 | 0.556 |
| UserPolitics:EngageIV | 0.045 | 0.078 | 0.573 | 0.567 |
| Concord:UserPolitics:EngageIV | -0.205 | 0.112 | -1.837 | 0.067 |
| Discord:UserPolitics:EngageIV | 0.044 | 0.105 | 0.423 | 0.673 |

**p*<.05, ***p*<.01, ****p*<.001

Note: Linear model predicting follow-back decision (1=follow, 0=block or ignore) by political concordance dummy, political discordance dummy (concordance and discordance for political preference = 4 determined via follow-up binary forced choice partisanship item), partisanship (*z*-scored), engagement condition (centered), and interactions between shared partisanship dummies, user partisanship, and engagement; filtering for passing at least 2 of 4 attention check items.

**Table S26: Blocking Predicted by User Partisanship, Account Partisanship, Engagement, and Twitter Usage.**

|  | ***b*** | ***SE*** | ***t*** | ***p*** |
| --- | --- | --- | --- | --- |
| (Intercept) | 0.044 | 0.018 | 2.391 | 0.017* |
| UserPolitics | 0.032 | 0.02 | 1.643 | 0.101 |
| DemBot | 0.015 | 0.027 | 0.543 | 0.587 |
| RepBot | 0.11 | 0.027 | 4.074 | <0.001*** |
| EngageIV | -0.018 | 0.037 | -0.496 | 0.62 |
| TwitPC | -0.02 | 0.029 | -0.706 | 0.48 |
| UserPolitics:DemBot | -0.041 | 0.027 | -1.506 | 0.133 |
| UserPolitics:RepBot | -0.098 | 0.028 | -3.43 | 0.001*** |
| UserPolitics:EngageIV | -0.031 | 0.039 | -0.806 | 0.42 |
| DemBot:EngageIV | -0.008 | 0.055 | -0.15 | 0.881 |
| RepBot:EngageIV | -0.032 | 0.054 | -0.594 | 0.553 |
| UserPolitics:TwitPC | -0.012 | 0.03 | -0.385 | 0.7 |
| DemBot:TwitPC | 0.014 | 0.04 | 0.358 | 0.72 |
| RepBot:TwitPC | -0.053 | 0.045 | -1.175 | 0.241 |
| EngageIV:TwitPC | 0.075 | 0.057 | 1.306 | 0.192 |
| UserPolitics:DemBot:EngageIV | 0.023 | 0.054 | 0.422 | 0.673 |
| UserPolitics:RepBot:EngageIV | 0.054 | 0.057 | 0.94 | 0.347 |
| UserPolitics:DemBot:TwitPC | 0.044 | 0.047 | 0.942 | 0.347 |
| UserPolitics:RepBot:TwitPC | 0.131 | 0.045 | 2.906 | 0.004** |
| UserPolitics:EngageIV:TwitPC | 0.138 | 0.06 | 2.305 | 0.021* |
| DemBot:EngageIV:TwitPC | -0.081 | 0.081 | -1.006 | 0.315 |
| RepBot:EngageIV:TwitPC | -0.115 | 0.09 | -1.268 | 0.205 |
| UserPolitics:DemBot:EngageIV:TwitPC | -0.174 | 0.094 | -1.846 | 0.065 |
| UserPolitics:RepBot:EngageIV:TwitPC | -0.228 | 0.09 | -2.531 | 0.012* |

**p*<.05, ***p*<.01, ****p*<.001

Note: Linear model predicting blocking decision (1=block, 0=follow or ignore) by user partisanship (1=Strongly Democratic, 7=Strongly Republican; *z*-scored), bot partisanship condition dummies (baseline = neutral control; dummies for Democratic Bot, Republican Bot), engagement condition (centered; -0.5=no engagement, 0.5=engagement), and the first principal component of five Twitter usage covariates (Twitter usage frequency, Twitter tweeting frequency, total number of Tweets, number of accounts followed, number of followers); allowing for all interactions.

**Table S27: Twitter UsagePCA First Component Loadings.**

|  | PC1 |
| --- | --- |
| Twitter_Frequency | 0.265 |
| Tweeting_Frequency | 0.301 |
| Total_Tweets | -0.003 |
| Total_Following | 0.646 |
| Total_Followers | 0.65 |

**Table S28: Blocking Predicted by Shared Partisanship, User Partisanship, Engagement, and Twitter Usage.**

|  | ***b*** | ***SE*** | ***t*** | ***p*** |
| --- | --- | --- | --- | --- |
| (Intercept) | 0.044 | 0.018 | 2.412 | 0.016* |
| Concord | 0.004 | 0.027 | 0.152 | 0.879 |
| Discord | 0.132 | 0.026 | 5.085 | <0.001*** |
| UserPolitics | 0.032 | 0.019 | 1.657 | 0.098 |
| EngageIV | -0.018 | 0.037 | -0.5 | 0.617 |
| TwitPC | -0.02 | 0.028 | -0.712 | 0.476 |
| Concord:UserPolitics | -0.056 | 0.029 | -1.952 | 0.051 |
| Discord:UserPolitics | -0.072 | 0.026 | -2.721 | 0.007** |
| Concord:EngageIV | -0.008 | 0.055 | -0.146 | 0.884 |
| Discord:EngageIV | -0.062 | 0.052 | -1.186 | 0.236 |
| UserPolitics:EngageIV | -0.031 | 0.039 | -0.813 | 0.416 |
| Concord:TwitPC | -0.036 | 0.047 | -0.763 | 0.446 |
| Discord:TwitPC | 0.02 | 0.039 | 0.513 | 0.608 |
| UserPolitics:TwitPC | -0.012 | 0.03 | -0.389 | 0.698 |
| EngageIV:TwitPC | 0.075 | 0.057 | 1.318 | 0.188 |
| Concord:UserPolitics:EngageIV | 0.014 | 0.057 | 0.248 | 0.805 |
| Discord:UserPolitics:EngageIV | 0.018 | 0.053 | 0.333 | 0.739 |
| Concord:UserPolitics:TwitPC | 0.027 | 0.046 | 0.584 | 0.56 |
| Discord:UserPolitics:TwitPC | 0.178 | 0.044 | 4.03 | <0.001*** |
| Concord:EngageIV:TwitPC | -0.086 | 0.093 | -0.92 | 0.358 |
| Discord:EngageIV:TwitPC | -0.145 | 0.077 | -1.879 | 0.061 |
| UserPolitics:EngageIV:TwitPC | 0.138 | 0.06 | 2.325 | 0.02* |
| Concord:UserPolitics:EngageIV:TwitPC | -0.134 | 0.093 | -1.447 | 0.148 |
| Discord:UserPolitics:EngageIV:TwitPC | -0.329 | 0.088 | -3.729 | <0.001*** |

**p*<.05, ***p*<.01, ****p*<.001

Note: Linear model predicting blocking decision (1=block, 0=follow or ignore) by political concordance dummy, political discordance dummy (concordance and discordance for political preference = 4 determined via follow-up binary forced choice partisanship item), partisanship (*z*-scored), engagement condition (centered), and the first principal component of five Twitter usage covariates (Twitter usage frequency, Twitter tweeting frequency, total number of Tweets, number of accounts followed, number of followers); allowing for all interactions.

**Table S29: Blocking Predicted by User Partisanship, Account Partisanship, and Engagement; Filtering for Blocking Knowledge (*N*=298).**

|  | ***b*** | ***SE*** | ***t*** | ***p*** |
| --- | --- | --- | --- | --- |
| (Intercept) | 0.042 | 0.028 | 1.468 | 0.143 |
| UserPolitics | 0.047 | 0.033 | 1.407 | 0.16 |
| DemBot | 0.011 | 0.041 | 0.274 | 0.785 |
| RepBot | 0.162 | 0.041 | 4.009 | <0.001*** |
| EngageIV | -0.045 | 0.057 | -0.796 | 0.427 |
| UserPolitics:DemBot | -0.057 | 0.044 | -1.294 | 0.197 |
| UserPolitics:RepBot | -0.157 | 0.046 | -3.404 | 0.001*** |
| UserPolitics:EngageIV | -0.016 | 0.067 | -0.243 | 0.808 |
| DemBot:EngageIV | 0.019 | 0.081 | 0.239 | 0.812 |
| RepBot:EngageIV | -0.102 | 0.081 | -1.265 | 0.207 |
| UserPolitics:DemBot:EngageIV | 0.017 | 0.087 | 0.2 | 0.842 |
| UserPolitics:RepBot:EngageIV | 0.082 | 0.092 | 0.891 | 0.374 |

**p*<.05, ***p*<.01, ****p*<.001

Note: Linear model predicting blocking decision (1=block, 0=follow or ignore) by user partisanship (1=Strongly Democratic, 7=Strongly Republican; *z*-scored), bot partisanship condition dummies (baseline = neutral control; dummies for Democratic Bot, Republican Bot), and engagement condition (centered; -0.5=no engagement, 0.5=engagement), allowing for all interactions; filtering for correctly answering a multiple choice question on how to block people on Twitter.

**Table S30: Blocking Predicted by Shared Partisanship, User Partisanship, and Engagement; Filtering for Blocking Knowledge (*N*=298).**

|  | ***b*** | ***SE*** | ***t*** | ***p*** |
| --- | --- | --- | --- | --- |
| (Intercept) | 0.042 | 0.028 | 1.478 | 0.141 |
| Concord | 0.00 | 0.041 | 0.006 | 0.996 |
| Discord | 0.176 | 0.04 | 4.445 | <0.001*** |
| UserPolitics | 0.047 | 0.033 | 1.416 | 0.158 |
| EngageIV | -0.045 | 0.057 | -0.801 | 0.424 |
| Concord:UserPolitics | -0.059 | 0.045 | -1.318 | 0.189 |
| Discord:UserPolitics | -0.152 | 0.044 | -3.457 | 0.001*** |
| Concord:EngageIV | 0.005 | 0.081 | 0.065 | 0.949 |
| Discord:EngageIV | -0.104 | 0.079 | -1.311 | 0.191 |
| UserPolitics:EngageIV | -0.016 | 0.066 | -0.245 | 0.807 |
| Concord:UserPolitics:EngageIV | 0.013 | 0.09 | 0.148 | 0.883 |
| Discord:UserPolitics:EngageIV | 0.086 | 0.088 | 0.973 | 0.331 |

**p*<.05, ***p*<.01, ****p*<.001

Note: Linear model predicting blocking decision (1=block, 0=follow or ignore) by political concordance dummy, political discordance dummy (concordance and discordance for political preference = 4 determined via follow-up binary forced choice partisanship item), partisanship (*z*-scored), engagement condition (centered), and interactions between shared partisanship dummies, user partisanship, and engagement; filtering for correctly answering a multiple choice question on how to block people on Twitter.

**Table S31: Blocking Predicted by User Partisanship, Account Partisanship, and Engagement; Filtering for Past RTing of MSNBC or Fox News (*N*=224).**

|  | ***b*** | ***SE*** | ***t*** | ***p*** |
| --- | --- | --- | --- | --- |
| (Intercept) | 0.026 | 0.023 | 1.159 | 0.248 |
| UserPolitics | 0.014 | 0.021 | 0.671 | 0.503 |
| DemBot | 0.012 | 0.031 | 0.389 | 0.698 |
| RepBot | 0.015 | 0.032 | 0.48 | 0.632 |
| EngageIV | -0.052 | 0.045 | -1.159 | 0.248 |
| UserPolitics:DemBot | 0.00 | 0.028 | -0.002 | 0.998 |
| UserPolitics:RepBot | -0.015 | 0.031 | -0.478 | 0.633 |
| UserPolitics:EngageIV | -0.028 | 0.042 | -0.671 | 0.503 |
| DemBot:EngageIV | 0.071 | 0.063 | 1.138 | 0.256 |
| RepBot:EngageIV | -0.031 | 0.064 | -0.48 | 0.632 |
| UserPolitics:DemBot:EngageIV | 0.033 | 0.057 | 0.584 | 0.56 |
| UserPolitics:RepBot:EngageIV | 0.029 | 0.061 | 0.478 | 0.633 |

**p*<.05, ***p*<.01, ****p*<.001

Note: Linear model predicting blocking decision (1=block, 0=follow or ignore) by user partisanship (1=Strongly Democratic, 7=Strongly Republican; *z*-scored), bot partisanship condition dummies (baseline = neutral control; dummies for Democratic Bot, Republican Bot), and engagement condition (centered; -0.5=no engagement, 0.5=engagement), allowing for all interactions; filtering for self-reported past retweeting of MSNBC or Fox News.

**Table S32: Blocking Predicted by Shared Partisanship, User Partisanship, and Engagement; Filtering for Past RTing of MSNBC or Fox News (*N*=224).**

|  | ***b*** | ***SE*** | ***t*** | ***p*** |
| --- | --- | --- | --- | --- |
| (Intercept) | 0.026 | 0.023 | 1.159 | 0.248 |
| Concord | -0.012 | 0.033 | -0.373 | 0.709 |
| Discord | 0.038 | 0.031 | 1.222 | 0.223 |
| UserPolitics | 0.014 | 0.021 | 0.671 | 0.503 |
| EngageIV | -0.052 | 0.045 | -1.159 | 0.248 |
| Concord:UserPolitics | -0.009 | 0.031 | -0.278 | 0.781 |
| Discord:UserPolitics | -0.004 | 0.028 | -0.15 | 0.881 |
| Concord:EngageIV | 0.024 | 0.066 | 0.373 | 0.709 |
| Discord:EngageIV | 0.009 | 0.062 | 0.139 | 0.89 |
| UserPolitics:EngageIV | -0.028 | 0.042 | -0.671 | 0.503 |
| Concord:UserPolitics:EngageIV | 0.017 | 0.062 | 0.278 | 0.781 |
| Discord:UserPolitics:EngageIV | 0.048 | 0.056 | 0.856 | 0.393 |

**p*<.05, ***p*<.01, ****p*<.001

Note: Linear model predicting blocking decision (1=block, 0=follow or ignore) by political concordance dummy, political discordance dummy (concordance and discordance for political preference = 4 determined via follow-up binary forced choice partisanship item), partisanship (*z*-scored), engagement condition (centered), and interactions between shared partisanship dummies, user partisanship, and engagement; filtering for self-reported past retweeting of MSNBC or Fox News.


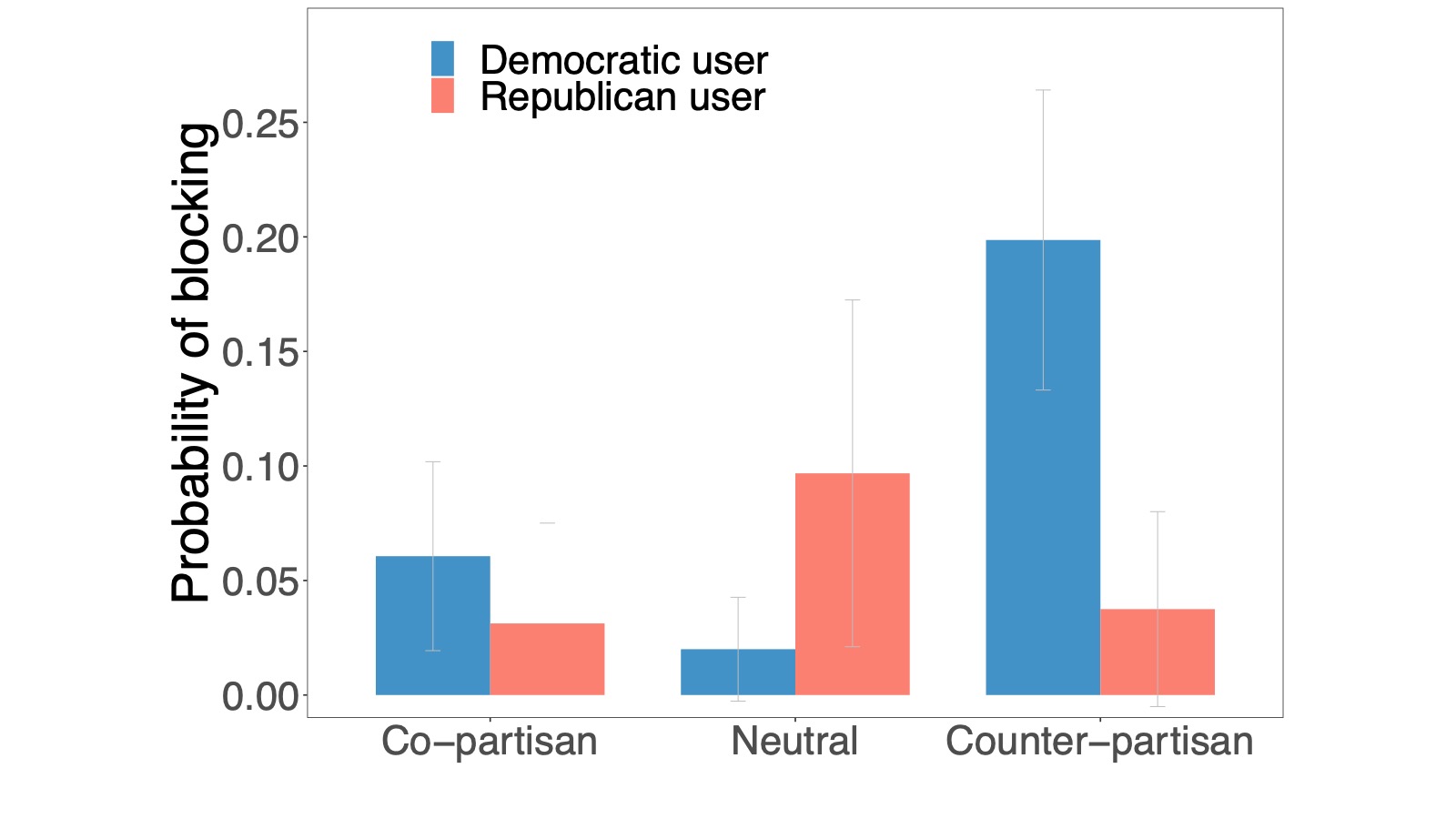


***Figure S2.*** *Probability of blocking by shared partisanship and user partisanship in Supplementary Survey Experiment 1. Error bars indicate 95% confidence intervals.*


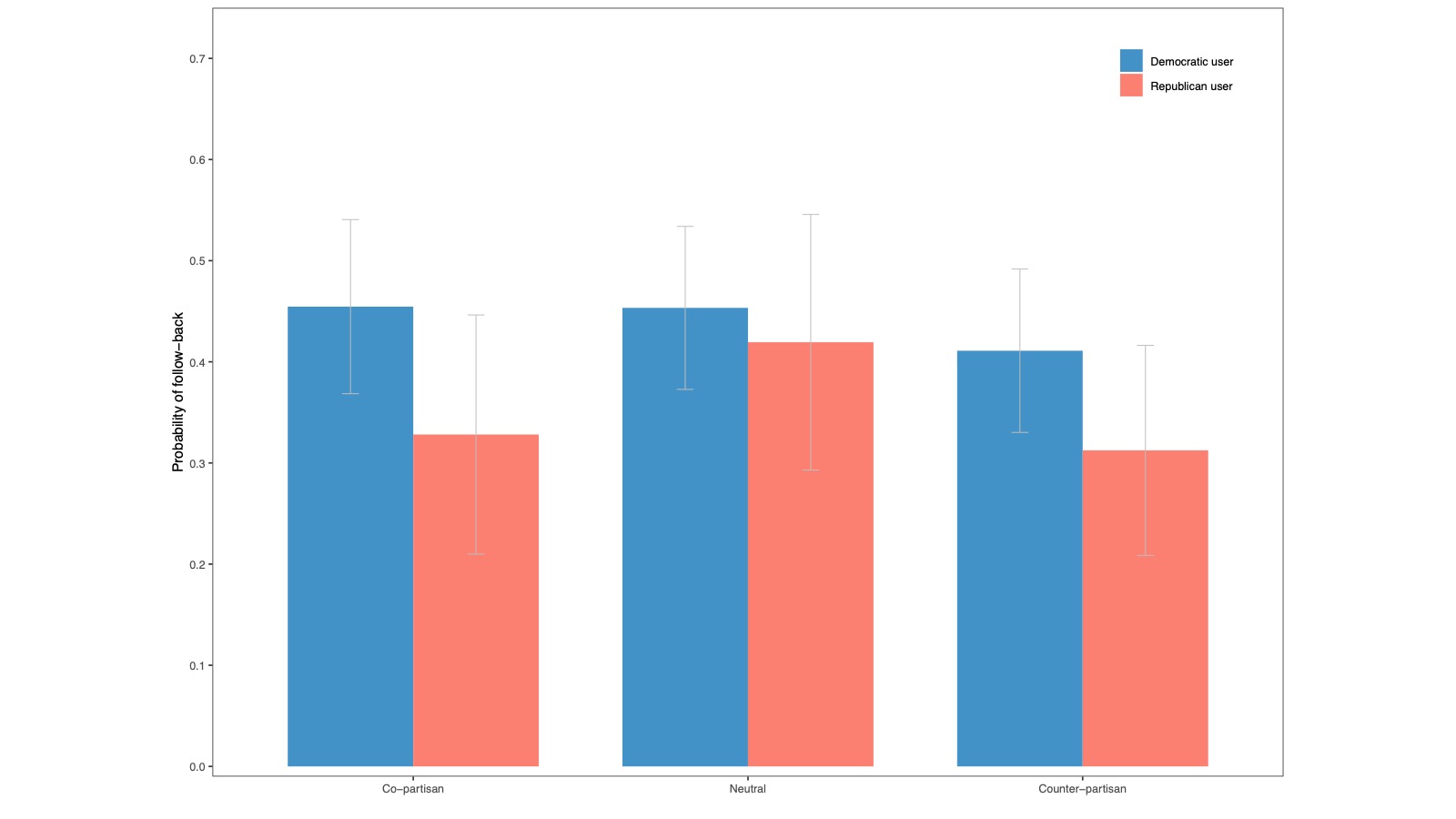


***Figure S3.*** *Probability of follow-back by shared partisanship and user partisanship in Supplementary Survey Experiment 1. Error bars indicate 95% confidence intervals.*

1. **Supplementary Survey Experiment 2.**

**Open Science Statement.**

Supplementary Survey Experiment 2 constituted the initial follow-up survey experiment to our field experiments. We pre-registered this study here: <https://aspredicted.org/blind.php?x=BSW_P5Y>. As in our main survey experiment and Supplementary Survey Experiment 1, we recruited participants on Lucid filtering for Twitter users, and adjusting Lucid’s recruitment quotas to better reflect a representative U.S. Twitter population sample by age, gender, and ethnicity.

The main differences between Supplementary Survey Experiment 2 and our other reported survey experiments are (a) we included an additional follower account condition to the Democrat, Republican, and neutral control - a ‘toxic’ profile (bio: ‘Professional troll | Hate speech is free speech | #FightMe), and (b) the multiple choice question for reasons why participants blocked was multi-select, rather than single-select.

We report all pre-registered analyses here. Three deviations in reported analyses are (i) we report descriptive statistics for blocking reasons, rather than multiple analyses per multiple choice option, due to data sparsity, (ii) we do not use natural language processing techniques or raters to evaluate the free-response items collected because of data sparsity, and (iii) as main analyses, we report a model using concordant and discordant shared partisanship dummy variables, as in our other survey experiment analyses - this additional model is included for easier comparison across studies.

Survey materials, data, and analysis files are available here: <https://osf.io/46aqr/?view_only=3a7f16b62131490aabb83a782fa133e5>.

**Table S33: Demographics.**

| ***N*** | 875 |
| --- | --- |
| **Gender** | 463 Female,  403 Male,  9 Other |
| **Age** | *M*_age_= 40.23 |
| **Race/Ethnicity** | 546 White (only) |

**Table S34: Blocking Predicted by User Partisanship, Account Partisanship, and Engagement.**

|  | ***b*** | ***SE*** | ***t*** | ***p*** |
| --- | --- | --- | --- | --- |
| (Intercept) | 0.064 | 0.025 | 2.504 | 0.012* |
| cUserPolitics | 0.014 | 0.014 | 1.033 | 0.302 |
| DemBot | 0.045 | 0.035 | 1.271 | 0.204 |
| RepBot | 0.042 | 0.038 | 1.108 | 0.268 |
| ToxicBot | 0.095 | 0.039 | 2.452 | 0.014* |
| EngageIV | 0.007 | 0.051 | 0.143 | 0.887 |
| cUserPolitics:DemBot | 0.018 | 0.02 | 0.939 | 0.348 |
| cUserPolitics:RepBot | -0.054 | 0.02 | -2.632 | 0.009** |
| cUserPolitics:ToxicBot | -0.064 | 0.021 | -2.971 | 0.003** |
| cUserPolitics:EngageIV | 0.00 | 0.028 | 0.005 | 0.996 |
| DemBot:EngageIV | 0.034 | 0.071 | 0.481 | 0.631 |
| RepBot:EngageIV | -0.052 | 0.075 | -0.697 | 0.486 |
| ToxicBot:EngageIV | 0.012 | 0.077 | 0.152 | 0.879 |
| cUserPolitics:DemBot:EngageIV | 0.011 | 0.039 | 0.275 | 0.784 |
| cUserPolitics:RepBot:EngageIV | -0.002 | 0.041 | -0.053 | 0.958 |
| cUserPolitics:ToxicBot:EngageIV | 0.064 | 0.043 | 1.48 | 0.139 |

**p*<.05, ***p*<.01, ****p*<.001

Note: Linear model predicting blocking decision (1=block, 0=follow or ignore) by user partisanship (1=Strongly Democratic, 6=Strongly Republican; centered), bot partisanship condition dummies (baseline = neutral control; dummies for Democratic Bot, Republican Bot, Toxic Bot), and engagement condition (centered; -0.5=no engagement, 0.5=engagement), allowing for all interactions.

**Table S35: Blocking Predicted by User Partisanship, Shared Partisanship, and Engagement.**

|  | ***b*** | ***SE*** | ***t*** | ***p*** |
| --- | --- | --- | --- | --- |
| (Intercept) | 0.108 | 0.016 | 6.746 | <0.001*** |
| UserPolitics | -0.007 | 0.016 | -0.415 | 0.678 |
| cConcord | -0.124 | 0.032 | -3.887 | <0.001*** |
| EngageIV | 0.00 | 0.032 | -0.007 | 0.994 |
| UserPolitics:cConcord | 0.009 | 0.032 | 0.277 | 0.782 |
| UserPolitics:EngageIV | 0.013 | 0.032 | 0.405 | 0.686 |
| cConcord:EngageIV | 0.053 | 0.064 | 0.823 | 0.411 |
| UserPolitics:cConcord:EngageIV | -0.024 | 0.063 | -0.38 | 0.704 |

**p*<.05, ***p*<.01, ****p*<.001

Note: Linear model predicting blocking decision (1=block, 0=follow or ignore) by user partisanship (1=Strongly Democratic, 6=Strongly Republican; *z*-scored), account shared partisanship (centered; using only the Democratic and Republican bots; -0.5=discordant, 0.5=concordant), and engagement condition (centered; -0.5=no engagement, 0.5=engagement), allowing for all interactions; subsetting on only the Democratic and Republican account conditions.

**Table S36: Blocking Predicted by User Partisanship, Shared Partisanship, and Engagement [Non Pre-registered Model].**

|  | ***b*** | ***SE*** | ***t*** | ***p*** |
| --- | --- | --- | --- | --- |
| (Intercept) | 0.092 | 0.012 | 7.643 | <0.001*** |
| Concord | -0.003 | 0.014 | -0.209 | 0.835 |
| Discord | 0.056 | 0.014 | 4.1 | <0.001*** |
| UserPolitics | 0.004 | 0.012 | 0.309 | 0.758 |
| EngageIV | 0.001 | 0.024 | 0.045 | 0.964 |
| Concord:UserPolitics | -0.012 | 0.014 | -0.849 | 0.397 |
| Discord:UserPolitics | -0.016 | 0.014 | -1.209 | 0.227 |
| Concord:EngageIV | 0.009 | 0.027 | 0.316 | 0.752 |
| Discord:EngageIV | -0.016 | 0.027 | -0.57 | 0.569 |
| UserPolitics):EngageIV | 0.009 | 0.024 | 0.39 | 0.697 |
| Concord:UserPolitics:EngageIV | 0.00 | 0.027 | 0.01 | 0.992 |
| Discord:UserPolitics:EngageIV | 0.012 | 0.027 | 0.444 | 0.657 |

**p*<.05, ***p*<.01, ****p*<.001

Note: Non-pre-registered linear model predicting blocking decision (1=block, 0=follow or ignore) by user partisanship (1=Strongly Democratic, 6=Strongly Republican; *z*-scored), concordant partisanship dummy, discordant partisanship dummy, and engagement condition (centered; -0.5=no engagement, 0.5=engagement), allowing for all interactions (not between shared partisanship dummies); omitting Toxic Bot condition.

**Table S37: Follow-back Predicted by User Partisanship, Account Partisanship, and Engagement.**

|  | ***b*** | ***SE*** | ***t*** | ***p*** |
| --- | --- | --- | --- | --- |
| (Intercept) | 0.559 | 0.039 | 14.385 | <0.001*** |
| cUserPolitics | -0.025 | 0.021 | -1.163 | 0.245 |
| DemBot | -0.188 | 0.054 | -3.484 | 0.001*** |
| RepBot | -0.072 | 0.057 | -1.252 | 0.211 |
| ToxicBot | -0.23 | 0.059 | -3.905 | <0.001*** |
| EngageIV | 0.15 | 0.078 | 1.924 | 0.055 |
| cUserPolitics:DemBot | -0.07 | 0.03 | -2.353 | 0.019* |
| cUserPolitics:RepBot | 0.031 | 0.031 | 0.99 | 0.322 |
| cUserPolitics:ToxicBot | -0.038 | 0.033 | -1.171 | 0.242 |
| cUserPolitics:EngageIV | 0.032 | 0.042 | 0.766 | 0.444 |
| DemBot:EngageIV | -0.114 | 0.108 | -1.051 | 0.294 |
| RepBot:EngageIV | -0.257 | 0.115 | -2.235 | 0.026* |
| ToxicBot:EngageIV | -0.094 | 0.118 | -0.795 | 0.427 |
| cUserPolitics:DemBot:EngageIV | 0.001 | 0.06 | 0.014 | 0.989 |
| cUserPolitics:RepBot:EngageIV | -0.173 | 0.063 | -2.773 | 0.006** |
| cUserPolitics:ToxicBot:EngageIV | -0.081 | 0.065 | -1.243 | 0.214 |

**p*<.05, ***p*<.01, ****p*<.001

Note: Linear model predicting follow-back decision (1=follow, 0=block or ignore) by user partisanship (1=Strongly Democratic, 6=Strongly Republican; centered), bot partisanship condition dummies (baseline = neutral control; dummies for Democratic Bot, Republican Bot, Toxic Bot), and engagement condition (centered; -0.5=no engagement, 0.5=engagement), allowing for all interactions.

**Table S38: Follow-back Predicted by User Partisanship, Shared Partisanship, and Engagement.**

|  | ***b*** | ***SE*** | ***t*** | ***p*** |
| --- | --- | --- | --- | --- |
| (Intercept) | 0.47 | 0.025 | 18.897 | <0.001*** |
| UserPolitics | -0.069 | 0.025 | -2.798 | 0.005** |
| cConcord | 0.162 | 0.05 | 3.256 | 0.001** |
| EngageIV | 0.02 | 0.05 | 0.394 | 0.694 |
| UserPolitics:cConcord | 0.064 | 0.049 | 1.296 | 0.196 |
| UserPolitics:EngageIV | -0.08 | 0.049 | -1.616 | 0.107 |
| cConcord:EngageIV | -0.211 | 0.099 | -2.126 | 0.034* |
| UserPolitics:cConcord:EngageIV | 0.009 | 0.098 | 0.093 | 0.926 |

**p*<.05, ***p*<.01, ****p*<.001

Note: Linear model predicting follow-back decision (1=follow, 0=block or ignore) by user partisanship (1=Strongly Democratic, 6=Strongly Republican; *z*-scored), account shared partisanship (centered; using only the Democratic and Republican bots; -0.5=discordant, 0.5=concordant), and engagement condition (centered; -0.5=no engagement, 0.5=engagement), allowing for all interactions; subsetting on only the Democratic and Republican account conditions.

**Table S39: Follow-back Predicted by User Partisanship, Shared Partisanship, and Engagement [Non Pre-registered Model].**

|  | ***b*** | ***SE*** | ***t*** | ***p*** |
| --- | --- | --- | --- | --- |
| (Intercept) | 0.502 | 0.02 | 24.601 | <0.001*** |
| Concord | -0.014 | 0.023 | -0.593 | 0.553 |
| Discord | -0.093 | 0.023 | -3.984 | <0.001*** |
| UserPolitics | -0.062 | 0.02 | -3.03 | 0.003** |
| EngageIV | 0.061 | 0.041 | 1.505 | 0.133 |
| Concord:UserPolitics | 0.001 | 0.023 | 0.036 | 0.971 |
| Discord:UserPolitics | -0.031 | 0.023 | -1.315 | 0.189 |
| Concord:EngageIV | -0.096 | 0.046 | -2.067 | 0.039* |
| Discord:EngageIV | 0 | 0.047 | -0.009 | 0.993 |
| UserPolitics):EngageIV | -0.034 | 0.041 | -0.831 | 0.406 |
| Concord:UserPolitics:EngageIV | -0.059 | 0.046 | -1.27 | 0.205 |
| Discord:UserPolitics:EngageIV | -0.066 | 0.046 | -1.432 | 0.153 |

**p*<.05, ***p*<.01, ****p*<.001

Note: Non-pre-registered linear model predicting follow-back decision (1=follow, 0=block or ignore) by user partisanship (1=Strongly Democratic, 6=Strongly Republican; *z*-scored), concordant partisanship dummy, discordant partisanship dummy, and engagement condition (centered; -0.5=no engagement, 0.5=engagement), allowing for all interactions (not between shared partisanship dummies); omitting Toxic Bot condition.

**Table S40: Blocking Predicted by User Partisanship, Account Partisanship, and Engagement; Filtering for Attention (*N*=701).**

|  | ***b*** | ***SE*** | ***t*** | ***p*** |
| --- | --- | --- | --- | --- |
| (Intercept) | 0.076 | 0.029 | 2.657 | 0.008** |
| cUserPolitics | 0.015 | 0.016 | 0.97 | 0.332 |
| DemBot | 0.05 | 0.041 | 1.21 | 0.227 |
| RepBot | 0.038 | 0.042 | 0.902 | 0.368 |
| ToxicBot | 0.096 | 0.044 | 2.181 | 0.03* |
| EngageIV | 0.016 | 0.057 | 0.273 | 0.785 |
| cUserPolitics:DemBot | 0.026 | 0.022 | 1.165 | 0.244 |
| cUserPolitics:RepBot | -0.056 | 0.023 | -2.463 | 0.014* |
| cUserPolitics:ToxicBot | -0.058 | 0.024 | -2.396 | 0.017* |
| cUserPolitics:EngageIV | 0.002 | 0.031 | 0.065 | 0.948 |
| DemBot:EngageIV | 0.035 | 0.082 | 0.422 | 0.674 |
| RepBot:EngageIV | -0.081 | 0.085 | -0.95 | 0.343 |
| ToxicBot:EngageIV | -0.023 | 0.088 | -0.264 | 0.792 |
| cUserPolitics:DemBot:EngageIV | 0.014 | 0.045 | 0.311 | 0.756 |
| cUserPolitics:RepBot:EngageIV | -0.014 | 0.046 | -0.301 | 0.764 |
| cUserPolitics:ToxicBot:EngageIV | 0.075 | 0.048 | 1.546 | 0.123 |

**p*<.05, ***p*<.01, ****p*<.001

Note: Linear model predicting blocking decision (1=block, 0=follow or ignore) by user partisanship (1=Strongly Democratic, 6=Strongly Republican; centered), bot partisanship condition dummies (baseline = neutral control; dummies for Democratic Bot, Republican Bot, Toxic Bot), and engagement condition (centered; -0.5=no engagement, 0.5=engagement), allowing for all interactions; filtering for passing at least one of two attention checks.

**Table S41: Blocking Predicted by User Partisanship, Shared Partisanship, and Engagement; Filtering for Attention (*N*=701).**

|  | ***b*** | ***SE*** | ***t*** | ***p*** |
| --- | --- | --- | --- | --- |
| (Intercept) | 0.118 | 0.018 | 6.448 | <0.001*** |
| UserPolitics | -0.001 | 0.018 | -0.064 | 0.949 |
| cConcord | -0.142 | 0.037 | -3.879 | <0.001*** |
| EngageIV | 0.001 | 0.037 | 0.03 | 0.976 |
| UserPolitics:cConcord | -0.004 | 0.036 | -0.101 | 0.92 |
| UserPolitics:EngageIV | 0.011 | 0.036 | 0.307 | 0.759 |
| cConcord:EngageIV | 0.08 | 0.073 | 1.097 | 0.273 |
| UserPolitics:cConcord:EngageIV | -0.007 | 0.072 | -0.094 | 0.925 |

**p*<.05, ***p*<.01, ****p*<.001

Note: Linear model predicting blocking decision (1=block, 0=follow or ignore) by user partisanship (1=Strongly Democratic, 6=Strongly Republican; *z*-scored), account shared partisanship (centered; using only the Democratic and Republican bots; -0.5=discordant, 0.5=concordant), and engagement condition (centered; -0.5=no engagement, 0.5=engagement), allowing for all interactions; subsetting on only the Democratic and Republican account conditions; filtering for passing at least one of two attention checks.

**Table S42: Blocking Predicted by User Partisanship, Shared Partisanship, and Engagement; Filtering for Attention (*N*=701) [Non Pre-registered Model].**

|  | ***b*** | ***SE*** | ***t*** | ***p*** |
| --- | --- | --- | --- | --- |
| (Intercept) | 0.107 | 0.017 | 6.433 | <0.001*** |
| Concord | -0.002 | 0.019 | -0.096 | 0.924 |
| Discord | 0.067 | 0.019 | 3.456 | 0.001*** |
| UserPolitics | -0.007 | 0.031 | -0.234 | 0.815 |
| EngageIV | -0.015 | 0.033 | -0.455 | 0.649 |
| Concord:UserPolitics | -0.024 | 0.035 | -0.675 | 0.50 |
| Discord:UserPolitics | -0.02 | 0.035 | -0.564 | 0.573 |
| Concord:EngageIV | 0.014 | 0.037 | 0.378 | 0.705 |
| Discord:EngageIV | -0.043 | 0.039 | -1.12 | 0.263 |
| UserPolitics):EngageIV | 0.062 | 0.062 | 0.996 | 0.32 |
| Concord:UserPolitics:EngageIV | -0.004 | 0.07 | -0.062 | 0.95 |
| Discord:UserPolitics:EngageIV | 0.06 | 0.071 | 0.843 | 0.40 |

**p*<.05, ***p*<.01, ****p*<.001

Note: Non-pre-registered linear model predicting blocking decision (1=block, 0=follow or ignore) by user partisanship (1=Strongly Democratic, 6=Strongly Republican; *z*-scored), concordant partisanship dummy, discordant partisanship dummy, and engagement condition (centered; -0.5=no engagement, 0.5=engagement), allowing for all interactions (not between shared partisanship dummies); omitting Toxic Bot condition; filtering for passing at least one of two attention checks.

**Table S43: Follow-back Predicted by User Partisanship, Account Partisanship, and Engagement; Filtering for Attention (*N*=701).**

|  | ***b*** | ***SE*** | ***t*** | ***p*** |
| --- | --- | --- | --- | --- |
| (Intercept) | 0.545 | 0.043 | 12.821 | <0.001*** |
| cUserPolitics | -0.029 | 0.023 | -1.24 | 0.215 |
| DemBot | -0.138 | 0.061 | -2.25 | 0.025* |
| RepBot | -0.03 | 0.063 | -0.483 | 0.63 |
| ToxicBot | -0.192 | 0.065 | -2.946 | 0.003** |
| EngageIV | 0.117 | 0.085 | 1.375 | 0.17 |
| cUserPolitics:DemBot | -0.074 | 0.033 | -2.238 | 0.026* |
| cUserPolitics:RepBot | 0.039 | 0.034 | 1.139 | 0.255 |
| cUserPolitics:ToxicBot | -0.006 | 0.036 | -0.179 | 0.858 |
| cUserPolitics:EngageIV | 0.001 | 0.046 | 0.021 | 0.984 |
| DemBot:EngageIV | -0.07 | 0.122 | -0.57 | 0.569 |
| RepBot:EngageIV | -0.287 | 0.126 | -2.271 | 0.023* |
| ToxicBot:EngageIV | -0.035 | 0.13 | -0.266 | 0.79 |
| cUserPolitics:DemBot:EngageIV | 0.057 | 0.066 | 0.859 | 0.39 |
| cUserPolitics:RepBot:EngageIV | -0.149 | 0.068 | -2.195 | 0.029* |
| cUserPolitics:ToxicBot:EngageIV | -0.072 | 0.072 | -0.995 | 0.32 |

**p*<.05, ***p*<.01, ****p*<.001

Note: Linear model predicting follow-back decision (1=follow, 0=block or ignore) by user partisanship (1=Strongly Democratic, 6=Strongly Republican; centered), bot partisanship condition dummies (baseline = neutral control; dummies for Democratic Bot, Republican Bot, Toxic Bot), and engagement condition (centered; -0.5=no engagement, 0.5=engagement), allowing for all interactions; filtering for passing at least one of two attention checks.

**Table S44: Follow-back Predicted by User Partisanship, Shared Partisanship, and Engagement; Filtering for Attention (*N*=701).**

|  | ***b*** | ***SE*** | ***t*** | ***p*** |
| --- | --- | --- | --- | --- |
| (Intercept) | 0.505 | 0.027 | 18.46 | <0.001*** |
| UserPolitics | -0.07 | 0.027 | -2.611 | 0.009** |
| cConcord | 0.206 | 0.055 | 3.773 | <0.001*** |
| EngageIV | -0.026 | 0.055 | -0.47 | 0.639 |
| UserPolitics:cConcord | 0.079 | 0.054 | 1.474 | 0.142 |
| UserPolitics:EngageIV | -0.072 | 0.054 | -1.342 | 0.181 |
| cConcord:EngageIV | -0.28 | 0.109 | -2.56 | 0.011* |
| UserPolitics:cConcord:EngageIV | -0.054 | 0.107 | -0.503 | 0.615 |

**p*<.05, ***p*<.01, ****p*<.001

Note: Linear model predicting follow-back decision (1=follow, 0=block or ignore) by user partisanship (1=Strongly Democratic, 6=Strongly Republican; *z*-scored), account shared partisanship (centered; using only the Democratic and Republican bots; -0.5=discordant, 0.5=concordant), and engagement condition (centered; -0.5=no engagement, 0.5=engagement), allowing for all interactions; subsetting on only the Democratic and Republican account conditions; filtering for passing at least one of two attention checks.

**Table S45: Follow-back Predicted by User Partisanship, Shared Partisanship, and Engagement; Filtering for Attention (*N*=701) [Non Pre-registered Model].**

|  | ***b*** | ***SE*** | ***t*** | ***p*** |
| --- | --- | --- | --- | --- |
| (Intercept) | 0.547 | 0.027 | 20.258 | <0.001*** |
| Concord | 0.02 | 0.03 | 0.667 | 0.505 |
| Discord | -0.044 | 0.032 | -1.406 | 0.16 |
| UserPolitics | -0.091 | 0.05 | -1.799 | 0.073 |
| EngageIV | 0.058 | 0.054 | 1.078 | 0.282 |
| Concord:UserPolitics | 0.003 | 0.057 | 0.061 | 0.951 |
| Discord:UserPolitics | -0.116 | 0.058 | -2.016 | 0.044* |
| Concord:EngageIV | -0.087 | 0.061 | -1.43 | 0.153 |
| Discord:EngageIV | -0.004 | 0.063 | -0.056 | 0.956 |
| UserPolitics):EngageIV | -0.11 | 0.101 | -1.092 | 0.276 |
| Concord:UserPolitics:EngageIV | -0.159 | 0.114 | -1.402 | 0.162 |
| Discord:UserPolitics:EngageIV | -0.016 | 0.115 | -0.14 | 0.889 |

**p*<.05, ***p*<.01, ****p*<.001

Note: Non-pre-registered linear model predicting follow-back decision (1=follow, 0=block or ignore) by user partisanship (1=Strongly Democratic, 6=Strongly Republican; *z*-scored), concordant partisanship dummy, discordant partisanship dummy, and engagement condition (centered; -0.5=no engagement, 0.5=engagement), allowing for all interactions (not between shared partisanship dummies); omitting Toxic Bot condition; filtering for passing at least one of two attention checks.

**Table S46: Blocking Predicted by User Partisanship, Account Partisanship, Engagement, and Twitter Usage.**

|  | ***b*** | ***SE*** | ***t*** | ***p*** |
| --- | --- | --- | --- | --- |
| (Intercept) | 0.061 | 0.028 | 2.183 | 0.029* |
| cUserPolitics | 0.012 | 0.014 | 0.854 | 0.394 |
| DemBot | 0.065 | 0.042 | 1.532 | 0.126 |
| RepBot | 0.037 | 0.042 | 0.884 | 0.377 |
| ToxicBot | 0.069 | 0.045 | 1.538 | 0.125 |
| EngageIV | 0.031 | 0.056 | 0.544 | 0.586 |
| TwitPC | 0.002 | 0.183 | 0.014 | 0.989 |
| cUserPolitics:DemBot | 0.037 | 0.022 | 1.71 | 0.088 |
| cUserPolitics:RepBot | -0.053 | 0.022 | -2.445 | 0.015* |
| cUserPolitics:ToxicBot | -0.067 | 0.023 | -2.908 | 0.004** |
| cUserPolitics:EngageIV | 0.009 | 0.029 | 0.322 | 0.748 |
| DemBot:EngageIV | 0.08 | 0.084 | 0.953 | 0.341 |
| RepBot:EngageIV | -0.072 | 0.084 | -0.862 | 0.389 |
| ToxicBot:EngageIV | -0.034 | 0.09 | -0.374 | 0.709 |
| cUserPolitics:TwitPC | -0.044 | 0.094 | -0.472 | 0.637 |
| DemBot:TwitPC | 0.051 | 0.275 | 0.184 | 0.854 |
| RepBot:TwitPC | -0.111 | 0.275 | -0.405 | 0.685 |
| ToxicBot:TwitPC | -0.412 | 0.308 | -1.337 | 0.182 |
| EngageIV:TwitPC | 0.31 | 0.366 | 0.848 | 0.397 |
| cUserPolitics:DemBot:EngageIV | 0.045 | 0.043 | 1.032 | 0.303 |
| cUserPolitics:RepBot:EngageIV | -0.016 | 0.043 | -0.363 | 0.717 |
| cUserPolitics:ToxicBot:EngageIV | 0.041 | 0.046 | 0.892 | 0.373 |
| cUserPolitics:DemBot:TwitPC | 0.219 | 0.145 | 1.515 | 0.13 |
| cUserPolitics:RepBot:TwitPC | 0.098 | 0.152 | 0.644 | 0.52 |
| cUserPolitics:ToxicBot:TwitPC | 0.341 | 0.163 | 2.098 | 0.036* |
| cUserPolitics:EngageIV:TwitPC | 0.207 | 0.187 | 1.106 | 0.269 |
| DemBot:EngageIV:TwitPC | 0.209 | 0.549 | 0.382 | 0.703 |
| RepBot:EngageIV:TwitPC | -0.23 | 0.55 | -0.418 | 0.676 |
| ToxicBot:EngageIV:TwitPC | -0.351 | 0.616 | -0.57 | 0.569 |
| cUserPolitics:DemBot:EngageIV:TwitPC | 0.267 | 0.29 | 0.924 | 0.356 |
| cUserPolitics:RepBot:EngageIV:TwitPC | -0.402 | 0.305 | -1.321 | 0.187 |
| cUserPolitics:ToxicBot:EngageIV:TwitPC | -0.617 | 0.325 | -1.898 | 0.058 |

**p*<.05, ***p*<.01, ****p*<.001

Note: Linear model predicting blocking decision (1=block, 0=follow or ignore) by user partisanship (1=Strongly Democratic, 6=Strongly Republican; centered), bot partisanship condition dummies (baseline = neutral control; dummies for Democratic Bot, Republican Bot, Toxic Bot), engagement condition (centered; -0.5=no engagement, 0.5=engagement), and the first principal component of five Twitter usage covariates (Twitter usage frequency, Twitter tweeting frequency, total number of Tweets, number of accounts followed, number of followers; reverse-coded); allowing for all interactions.

**Table S47: Twitter UsagePCA First Component Loadings.**

|  | PC1 |
| --- | --- |
| Twitter_Frequency | -0.065 |
| Tweeting_Frequency | -0.073 |
| Total_Tweets | -0.575 |
| Total_Following | -0.575 |
| Total_Followers | -0.575 |

**Table S48: Blocking Predicted by User Partisanship, Shared Partisanship, Engagement, and Twitter Usage.**

|  | ***b*** | ***SE*** | ***t*** | ***p*** |
| --- | --- | --- | --- | --- |
| (Intercept) | 0.083 | 0.013 | 6.205 | <0.001*** |
| UserPolitics | 0.007 | 0.013 | 0.53 | 0.596 |
| cConcord | -0.151 | 0.033 | -4.523 | <0.001*** |
| EngageIV | 0.008 | 0.027 | 0.308 | 0.758 |
| TwitPC | -0.125 | 0.086 | -1.452 | 0.147 |
| UserPolitics:cConcord | -0.013 | 0.033 | -0.397 | 0.692 |
| UserPolitics:EngageIV | 0.024 | 0.025 | 0.955 | 0.34 |
| cConcord:EngageIV | -0.023 | 0.067 | -0.345 | 0.73 |
| UserPolitics:TwitPC | 0.07 | 0.088 | 0.803 | 0.422 |
| cConcord:TwitPC | -0.226 | 0.208 | -1.086 | 0.278 |
| EngageIV:TwitPC | -0.031 | 0.172 | -0.179 | 0.858 |
| UserPolitics:cConcord:EngageIV | -0.073 | 0.066 | -1.108 | 0.268 |
| UserPolitics:cConcord:TwitPC | -0.16 | 0.232 | -0.69 | 0.49 |
| UserPolitics:EngageIV:TwitPC | 0.273 | 0.175 | 1.556 | 0.12 |
| cConcord:EngageIV:TwitPC | -0.709 | 0.417 | -1.701 | 0.09 |
| UserPolitics:cConcord:EngageIV:TwitPC | -0.415 | 0.464 | -0.895 | 0.371 |

**p*<.05, ***p*<.01, ****p*<.001

Note: Linear model predicting blocking decision (1=block, 0=follow or ignore) by user partisanship (1=Strongly Democratic, 6=Strongly Republican; *z*-scored), account shared partisanship (centered; using only the Democratic and Republican bots; -0.5=discordant, 0.5=concordant), and engagement condition (centered; -0.5=no engagement, 0.5=engagement), and the first principal component of five Twitter usage covariates (Twitter usage frequency, Twitter tweeting frequency, total number of Tweets, number of accounts followed, number of followers; reverse-coded), allowing for all interactions; subsetting on only the Democratic and Republican account conditions.

**Table S49: Blocking Counts & Reasons (Multi-Select) by User-Account Matching.**

| **User - Account** | **Proportion Block** | **Never Want Their Content** | **Never Want Them to See My Content** | **Agree Politically** | **Disagree Politically** | **Never Want To Engage** | **Never Want To Argue** | **Do Not Want to Troll Them** | **Do Not Want to Troll Me** | **Likely A Bot** |
| --- | --- | --- | --- | --- | --- | --- | --- | --- | --- | --- |
| Dem-Rep | 0.177  (26/147) | 22 | 12 | 0 | 21 | 19 | 12 | 4 | 18 | 7 |
| Dem-Dem | 0.053  (7/132) | 3 | 1 | 0 | 2 | 2 | 0 | 1 | 3 | 1 |
| Dem-Neu | 0.046  (6/130) | 2 | 1 | 0 | 1 | 3 | 1 | 2 | 2 | 3 |
| Dem-Toxic | 0.242  (37/153) | 28 | 8 | 0 | 17 | 21 | 17 | 9 | 20 | 8 |
| Rep-Rep | 0.023  (1/44) | 1 | 1 | 0 | 0 | 1 | 1 | 0 | 1 | 1 |
| Rep-Dem | 0.147  (10/68) | 4 | 0 | 0 | 7 | 3 | 1 | 1 | 4 | 2 |
| Rep-Neu | 0.067  (4/60) | 1 | 2 | 0 | 0 | 2 | 0 | 1 | 1 | 0 |
| Rep-Toxic | 0.075  (3/40) | 3 | 2 | 0 | 1 | 3 | 2 | 1 | 2 | 1 |
| **Total** | — | **64** | **27** | **0** | **49** | **54** | **34** | **19** | **51** | **23** |


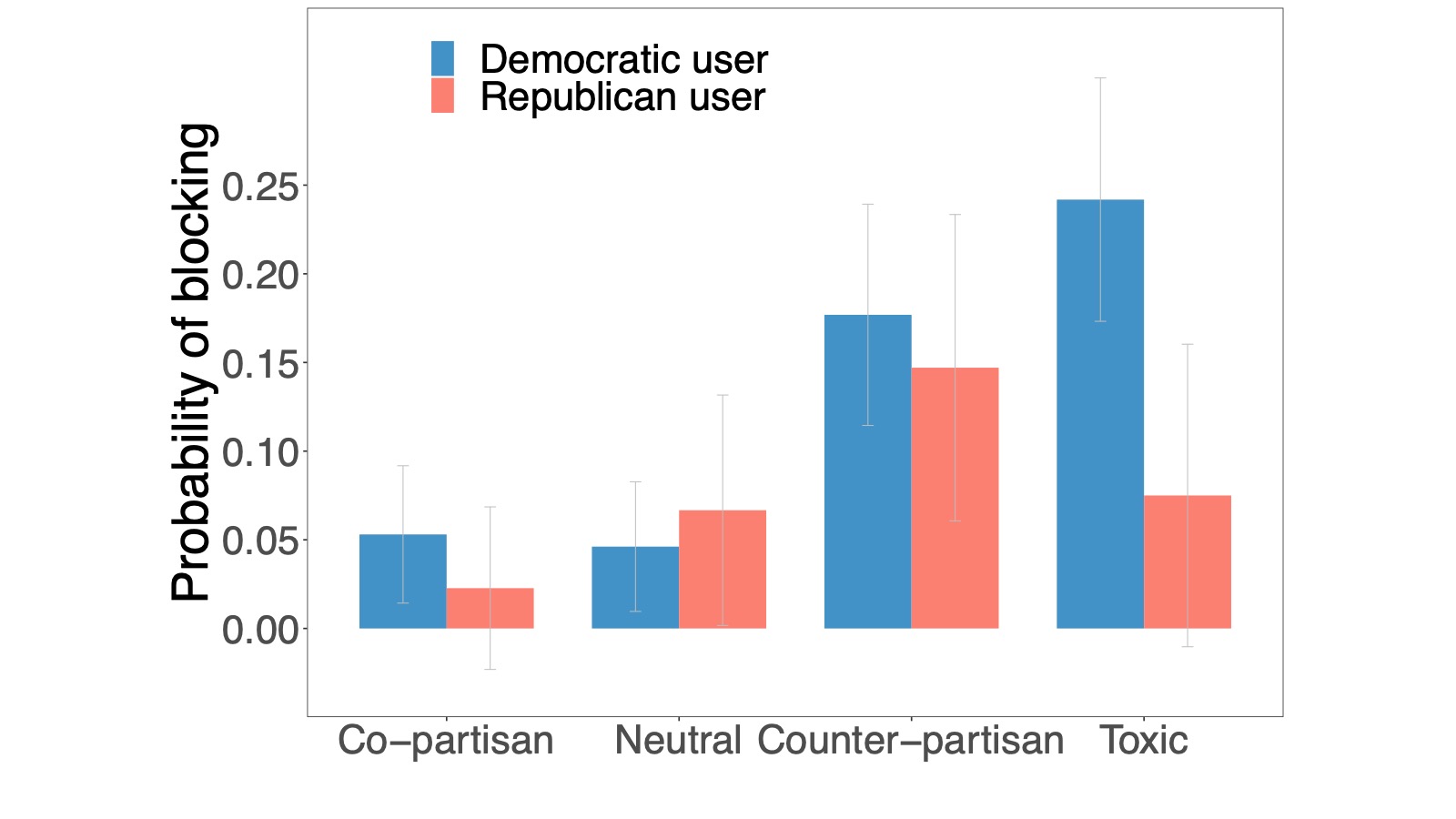


***Figure S4.*** *Probability of blocking by shared partisanship and user partisanship in Supplementary Survey Experiment 2. Error bars indicate 95% confidence intervals.*


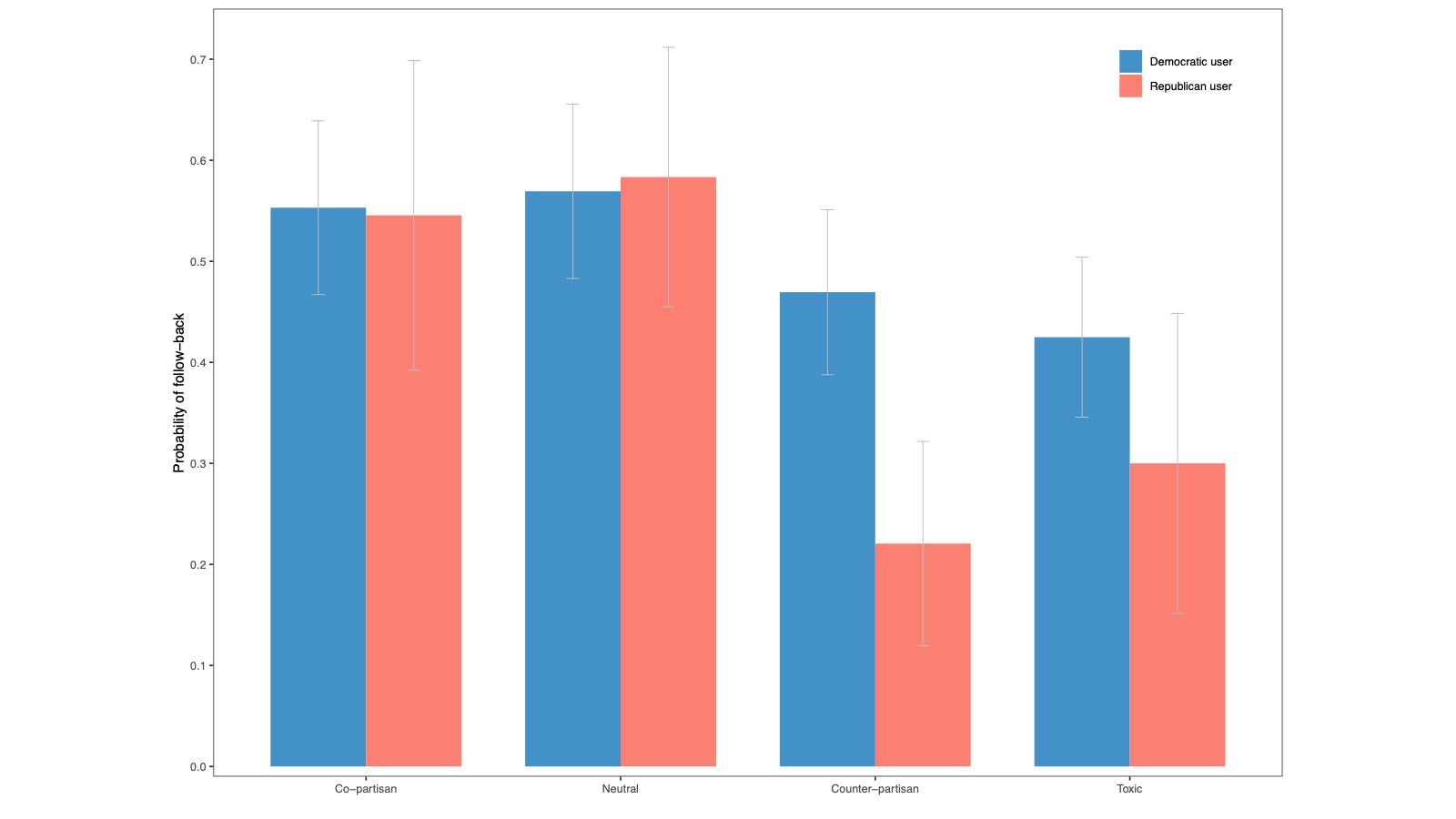


***Figure S5.*** *Probability of follow-back by shared partisanship and user partisanship in Supplementary Survey Experiment 2. Error bars indicate 95% confidence intervals.*

1. **Blocking Partisan Differences - Grid Item.**

**Open Science Statement.**

The partisan differences in blocking grid item was fielded as an additional measure in an unrelated study. The main task of this study was evaluating either accuracy or sharing intentions for a series of true and false news items. Participants received our blocking item following this task. We pre-registered analyzing these grid items in the secondary analyses section here: <https://aspredicted.org/blind.php?x=HNR_DBR>. Participants (*N*=3,057) were recruited from Lucid using standard nationally representative quotas.

In a grid, participants were asked the following: “On social media, how likely are you to mute, block, or unfollow people who share content that:” (1=Very unlikely, 2=Unlikely, 3=Neutral, 4=Likely, 5=Very likely). All participants received the following items, in random order: “You think is inaccurate or false”; “Is mean or nasty”; “Is racist, sexist, or contains hate speech”; “Is ‘woke’ or engages in cancel culture”; “Questions or doubts the existence of God.” Additionally, all participants received one political item - this item specified either criticism of the participants’ ingroup or praise of the participants’ outgroup (based on partisanship, assessed 1=Strongly Democratic, 6=Strongly Republican); and also varied whether it was referring to a specific political elite (Donald Trump, Joe Biden) or partisans in general (Republicans, Democrats). Democratic participants received one of the following: “Praises Donald Trump”; “Praises Republicans”; “Criticizes Joe Biden”; “Criticizes Democrats.” Republican participants received one of the following: “Praises Joe Biden”; “Praises Democrats”; “Criticizes Donald Trump”; “Criticizes Republicans.”

Survey materials, data, and analysis files are available here: <https://osf.io/46aqr/?view_only=3a7f16b62131490aabb83a782fa133e5>.

**Table S50: Blocking People Who Share False/Inaccurate Content by Partisanship**

|  | ***b*** | ***SE*** | ***t*** | ***p*** |
| --- | --- | --- | --- | --- |
| (Intercept) | 3.565 | 0.022 | 161.131 | <0.001*** |
| UserPolitics | -0.075 | 0.022 | -3.401 | 0.001*** |

**p*<.05, ***p*<.01, ****p*<.001

Note: Linear model predicting blocking likelihood (1=Very unlikely, 5=Very likely) by user partisanship (1=Strongly Democratic, 6=Strongly Republican; *z*-scored).

**Table S51: Blocking People Who Share Mean/Nasty Content by Partisanship**

|  | ***b*** | ***SE*** | ***t*** | ***p*** |
| --- | --- | --- | --- | --- |
| (Intercept) | 3.824 | 0.022 | 170.211 | <0.001*** |
| UserPolitics | -0.083 | 0.023 | -3.702 | <0.001*** |

**p*<.05, ***p*<.01, ****p*<.001

Note: Linear model predicting blocking likelihood (1=Very unlikely, 5=Very likely) by user partisanship (1=Strongly Democratic, 6=Strongly Republican; *z*-scored).

**Table S52: Blocking People Who Share Racist/Sexist/Hate Speech Content by Partisanship**

|  | ***b*** | ***SE*** | ***t*** | ***p*** |
| --- | --- | --- | --- | --- |
| (Intercept) | 3.965 | 0.023 | 175.920 | <0.001*** |
| UserPolitics | -0.153 | 0.023 | -6.762 | <0.001*** |

**p*<.05, ***p*<.01, ****p*<.001

Note: Linear model predicting blocking likelihood (1=Very unlikely, 5=Very likely) by user partisanship (1=Strongly Democratic, 6=Strongly Republican; *z*-scored).

**Table S53: Blocking People Who Share “Woke”/Cancel Culture Content by Partisanship**

|  | ***b*** | ***SE*** | ***t*** | ***p*** |
| --- | --- | --- | --- | --- |
| (Intercept) | 3.377 | 0.022 | 151.446 | <0.001*** |
| UserPolitics | 0.210 | 0.022 | 9.388 | <0.001*** |

**p*<.05, ***p*<.01, ****p*<.001

Note: Linear model predicting blocking likelihood (1=Very unlikely, 5=Very likely) by user partisanship (1=Strongly Democratic, 6=Strongly Republican; *z*-scored).

**Table S54: Blocking People Who Share Content Questioning/Doubting God by Partisanship**

|  | ***b*** | ***SE*** | ***t*** | ***p*** |
| --- | --- | --- | --- | --- |
| (Intercept) | 2.976 | 0.024 | 121.972 | <0.001*** |
| UserPolitics | 0.156 | 0.024 | 6.403 | <0.001*** |

**p*<.05, ***p*<.01, ****p*<.001

Note: Linear model predicting blocking likelihood (1=Very unlikely, 5=Very likely) by user partisanship (1=Strongly Democratic, 6=Strongly Republican; *z*-scored).

**Table S55: Blocking People Who Criticize In-party Elite/Partisans or Praise Out-party Elite/Partisans**

|  | ***b*** | ***SE*** | ***t*** | ***p*** |
| --- | --- | --- | --- | --- |
| (Intercept) | 3.121 | 0.024 | 132.722 | <0.001*** |
| UserPolitics | -0.018 | 0.024 | -0.743 | 0.458 |
| Criticize/Praise | -0.277 | 0.047 | -5.892 | <0.001*** |
| Elite/Partisans | 0.066 | 0.047 | 1.413 | 0.158 |
| UserPolitics X Criticize/Praise | 0.162 | 0.047 | 3.444 | 0.001*** |
| UserPolitics X Elite/Partisans | -0.036 | 0.047 | -0.77 | 0.442 |
| Criticize/Praise X Elite/Partisans | -0.31 | 0.094 | -3.296 | 0.001*** |
| UserPolitics X Criticize/Praise X Elite/Partisans | 0.12 | 0.094 | 1.268 | 0.205 |

**p*<.05, ***p*<.01, ****p*<.001

Note: Linear model predicting blocking likelihood (1=Very unlikely, 5=Very likely) by user partisanship (1=Strongly Democratic, 6=Strongly Republican; *z*-scored), criticizing in-party vs. praising outparty (centered; praise=-0.5, criticize=0.5), elite vs. party (centered; party=-0.5, elite=0.5), and all interactions.


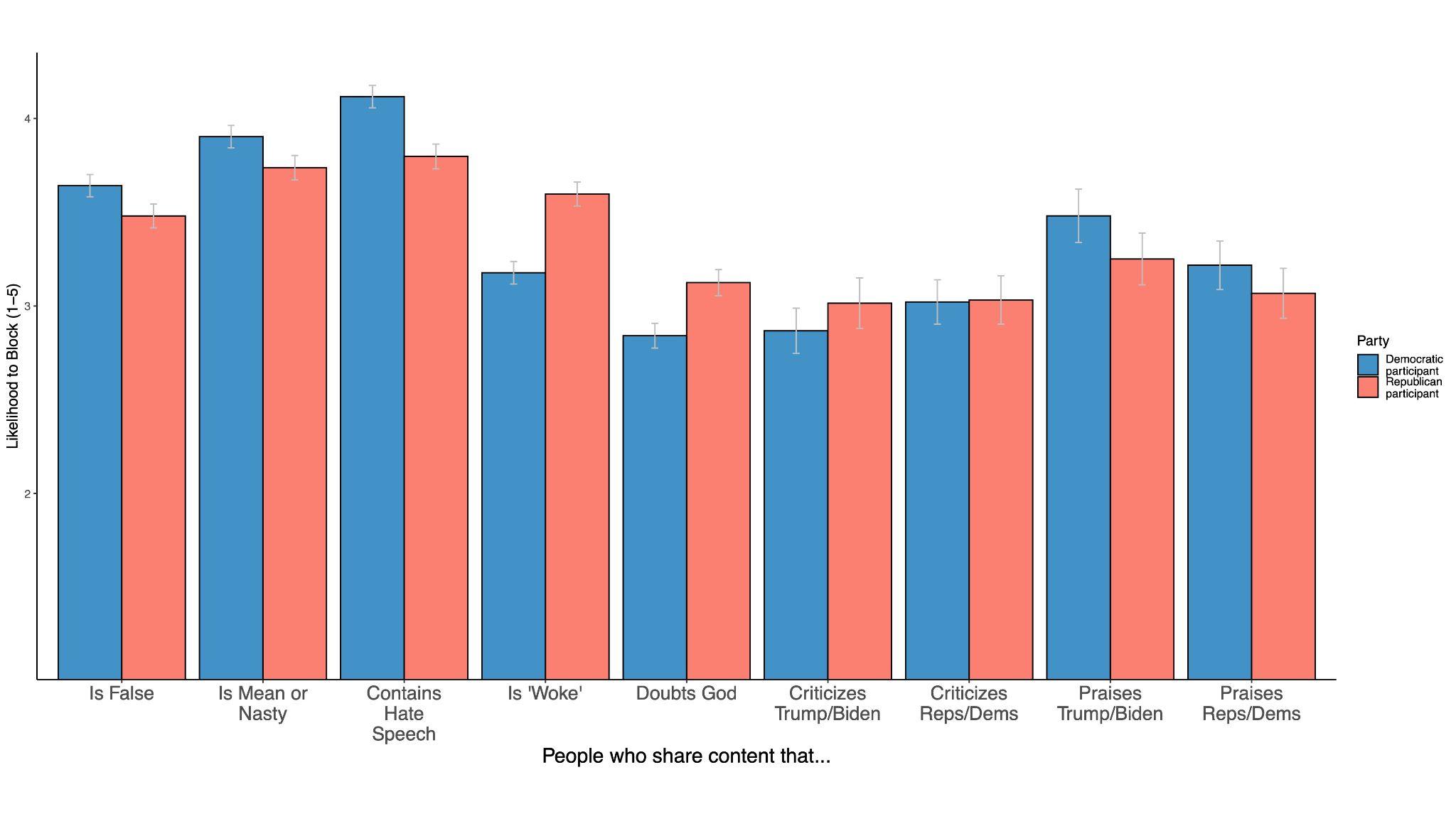


***Figure S6.*** *Likelihood of blocking people who share certain types of content by participant partisanship in supplementary grid item. Error bars indicate 95% confidence intervals.*

1. **Partisan Bot Content in Twitter Field Experiments.**

**Table S56: Quality of Content Retweeted by Partisan Bots in Field Experiment 1**

|  | ***b*** | ***SE*** | ***t*** | ***p*** |
| --- | --- | --- | --- | --- |
| (Intercept) | 0.273 | 0.000 | 1.13e+18 | <0.001*** |
| RepublicanBot | -0.473 | 0.011 | -43.175 | <0.001*** |

**p*<.05, ***p*<.01, ****p*<.001

Note: Linear model predicting retweeted news quality (z-scored; (37)) by bot partisanship with robust standard errors clustered on bot.

**Table S57: Partisan Slant of Content Retweeted by Partisan Bots in Field Experiment 1**

|  | ***b*** | ***SE*** | ***t*** | ***p*** |
| --- | --- | --- | --- | --- |
| (Intercept) | -0.908 | 0.000 | -1.439e+18 | <0.001*** |
| RepublicanBot | 1.787 | 0.069 | 25.839 | <0.001*** |

**p*<.05, ***p*<.01, ****p*<.001

Note: Linear model predicting retweeted news partisan slant (z-scored absolute value; (38)) by bot partisanship with robust standard errors clustered on bot.

**Table S58: Toxicity of Content Retweeted by Partisan Bots in Field Experiment 1**

|  | ***b*** | ***SE*** | ***t*** | ***p*** |
| --- | --- | --- | --- | --- |
| (Intercept) | -0.050 | 0.000 | 1.071e-144 | <0.001*** |
| RepublicanBot | 0.118 | 0.008 | 15.335 | <0.001*** |

**p*<.05, ***p*<.01, ****p*<.001

Note: Linear model predicting retweeted news toxicity (z-scored; Google Perspective API [<https://perspectiveapi.com/>]) by bot partisanship with robust standard errors clustered on bot.

**Table S59: Quality of Content Retweeted by Partisan Bots in Field Experiment 2**

|  | ***b*** | ***SE*** | ***t*** | ***p*** |
| --- | --- | --- | --- | --- |
| (Intercept) | -0.230 | 0.000 | -3.55e+15 | <0.001*** |
| NeutralBot | 1.151 | 0.000 | 5.45e+15 | <0.001*** |
| RepublicanBot | -0.639 | 0.000 | -1.13e+15 | <0.001*** |

**p*<.05, ***p*<.01, ****p*<.001

Note: Linear model predicting retweeted news quality (z-scored; (37)) by bot partisanship (baseline=Democratic bot) with robust standard errors clustered on bot.

**Table S60: Partisan Slant of Content Retweeted by Partisan Bots in Field Experiment 2**

|  | ***b*** | ***SE*** | ***t*** | ***p*** |
| --- | --- | --- | --- | --- |
| (Intercept) | -0.244 | 0.000 | 4.1e-29 | <0.001*** |
| NeutralBot | -0.397 | 0.000 | 1.57e-29 | <0.001*** |
| RepublicanBot | 1.492 | 0.000 | 2.29e-30 | <0.001*** |

**p*<.05, ***p*<.01, ****p*<.001

Note: Linear model predicting retweeted news partisan slant (z-scored absolute value; (38)) by bot partisanship (baseline=Democratic bot) with robust standard errors clustered on bot.

**Table S61: Toxicity of Content Retweeted by Partisan Bots in Field Experiment 2**

|  | ***b*** | ***SE*** | ***t*** | ***p*** |
| --- | --- | --- | --- | --- |
| (Intercept) | -0.009 | 0.000 | 1.28e-28 | <0.001*** |
| NeutralBot | -0.170 | 0.000 | 1.04e-30 | <0.001*** |
| RepublicanBot | 0.196 | 0.000 | 9.45e-31 | <0.001*** |

**p*<.05, ***p*<.01, ****p*<.001

Note: Linear model predicting retweeted news toxicity (z-scored; Google Perspective API [<https://perspectiveapi.com/>]) by bot partisanship (baseline=Democratic bot) with robust standard errors clustered on bot.

1. **Field Experiment 1 Analyses.**

**Table S62: Blocking Predicted by Discordance, User Partisanship, and their Interaction.**

|  | ***b*** | ***SE*** | ***t*** | ***p*** |
| --- | --- | --- | --- | --- |
| (Intercept) | 0.005 | 0.006 | 0.905 | 0.366 |
| Discord | 0.057 | 0.008 | 7.247 | <0.001*** |
| Republican_Dummy | 0.002 | 0.006 | 0.397 | 0.691 |
| Discord:Republican_Dummy | -0.031 | 0.008 | -3.893 | <0.001*** |

**p*<.05, ***p*<.01, ****p*<.001

Note: Linear model predicting blocking decision (1=block, 0=not block) by discordance between user and bot account (dummy), user partisanship (inferred partisanship from (44); *z*-scored dummy), and their interaction.

**Table S63: Follow-back Predicted by Discordance, User Partisanship, and their Interaction.**

|  | ***b*** | ***SE*** | ***t*** | ***P*** |
| --- | --- | --- | --- | --- |
| (Intercept) | 0.076 | 0.007 | 10.901 | <0.001*** |
| Discord | -0.048 | 0.010 | -4.905 | <0.001*** |
| zRepublican_Dummy | 0.002 | 0.007 | 0.227 | 0.821 |
| Discord:zRepublican_Dummy | -0.000 | 0.010 | -0.029 | 0.977 |

**p*<.05, ***p*<.01, ****p*<.001

Note: Linear model predicting follow-back decision (1=follow-back, 0=not follow-back) by discordance between user and bot account (dummy), user partisanship (inferred partisanship from (44); *z*-scored dummy), and their interaction.

1. **Field Experiment 2 Analyses.**

**Table S64: Blocking Predicted by Discordance, Concordance, User Partisanship, and their Interactions.**

|  | ***b*** | ***SE*** | ***t*** | ***p*** |
| --- | --- | --- | --- | --- |
| (Intercept) | 0.021 | 0.005 | 3.943 | <0.001*** |
| Discord | 0.068 | 0.008 | 8.993 | <0.001*** |
| Concord | -0.005 | 0.008 | -0.696 | 0.487 |
| zRepublican_Dummy | -0.011 | 0.005 | -2.026 | 0.043 |
| Discord:zRepublican_Dummy | -0.042 | 0.008 | -5.486 | <0.001*** |
| Concord:zRepublican_Dummy | 0.004 | 0.008 | 0.543 | 0.587 |

**p*<.05, ***p*<.01, ****p*<.001

Note: Linear model predicting blocking decision (1=block, 0=not block) by discordance between user and bot account (dummy), concordance between user and bot account (dummy), user partisanship (inferred partisanship from (44); *z*-scored dummy), and their interactions.

**Table S65: Follow-back Predicted by Discordance, Concordance, User Partisanship, and their Interactions.**

|  | ***b*** | ***SE*** | ***t*** | ***p*** |
| --- | --- | --- | --- | --- |
| (Intercept) | 0.118 | 0.008 | 13.907 | <0.001*** |
| Discord | -0.072 | 0.012 | -6.013 | <0.001*** |
| Concord | 0.055 | 0.012 | 4.582 | <0.001*** |
| zRepublican_Dummy | 0.015 | 0.008 | 1.758 | 0.079 |
| Discord:zRepublican_Dummy | 0.004 | 0.012 | 0.32 | 0.749 |
| Concord:zRepublican_Dummy | 0.008 | 0.012 | 0.701 | 0.483 |

**p*<.05, ***p*<.01, ****p*<.001

Note: Linear model predicting follow-back decision (1=follow-back, 0=not follow-back) by discordance between user and bot account (dummy), concordance between user and bot account (dummy), user partisanship (inferred partisanship from (44); *z*-scored dummy), and their interactions.
